# Pricing and service effort strategy in live streaming commerce supply chain under the equal proportion settlement mode

**DOI:** 10.1371/journal.pone.0309371

**Published:** 2024-08-29

**Authors:** Jinrong Liu, Yanfen Zhang, Zhongmiao Sun

**Affiliations:** 1 School of Economics and Trade, Shanghai Urban Construction Vocational College, Shanghai, China; 2 Glorious Sun School of Business and Management, Donghua University, Shanghai, China; 3 School of Economics and Management, Shanghai Maritime University, Shanghai, China; SEGi University Kota Damansara, MALAYSIA

## Abstract

Investigating novel collaboration modes is imperative for enhancing the cooperative ties between brand retailers and anchors as live streaming commerce moves progressively towards standardization. In this paper, we study a live streaming commerce supply chain system composed of a brand retailer and an Internet celebrity anchor. We also develop the pricing and live streaming service effort decision models under the equal proportion settlement mode of pit fee for the brand retailer and anchor. The research results show that under certain conditions, there is an optimal joint decision on price discount and live streaming service effort. In addition, the equal proportion settlement mode provides security for brand retailers but tends to expose the anchor’s capabilities of live streaming sales. Consequently, anchors can only obtain a higher proportion of the pit fee by putting in more effort. Finally, through numerical analysis, we presented the impact of key parameters on the optimal decisions, demand, and profits of brand retailers and anchors. Based on these findings, we provided relevant managerial insights, offering valuable guidance and reference for the live streaming commerce industry.

## 1. Introduction

The development of e-commerce is undergoing a significant evolution through the integration of various emerging novel applications aimed at enhancing customer engagement and achieving greater economic value [1]. As an interactive multimedia platform providing entertainment, social and commercial activities, live streaming has gained in popularity since 2011 [2]. This innovative form of live streaming commerce has transformed e-commerce from a product-oriented shopping environment into a social, hedonic, and customer-centered experience [3, 4]. Furthermore, live streaming selling has become one of the preferred channels for major vendors to increase product sales and clear inventory [5]. The rapid growth of the live streaming industry has increased the number of consumer options for purchase channels, which has had a significant impact on firm sales models and management practices [6].

In recent years, the live streaming (LS) industry has become a crucial platform for enhancing economic activity. Through the live streaming shopping function, sellers can use real-video to present products and interact with potential buyers. Consumers can also express their opinions and post their comments on a real-time basis [7]. In China, a sea of savvy business owners is turning to live streaming platforms such as Douyin (the equivalent of TikTok in China) and Kuaishou, as well as e-commerce giant Alibaba’s Taobao, to promote and sell their products in real time [8]. According to the 53rd statistical report of CNNIC, as of December 2023, the number of online live streaming (LS) users in China reached 816 million, accounting for 74.7% of the total Internet users. In 2023, the size of China’s live streaming commerce market was 4.9 trillion yuan. According to Star Map data, from 20:00 on November 10, 2023, to 24:00 on November 11, 2023, the comprehensive e-commerce GMV (Gross Merchandise Volume) reached approximately 277.7 billion yuan. From November 1 to November 10, the GMV of LS commerce platforms was 215.067 billion yuan, a year-on-year growth of approximately 18.6%, with platform GMV rankings of Douyin, Kuaishou, and Diantao. E-Commerce Daily announced that on October 25, 2023, the first day of the presale for Tmall’s Double 11, the GMV of Li Jiaqi, the top lipstick influencer, was up to 9.5 billion yuan.

However, in the strong wind of the live streaming commerce, not all brand retailers who entrust stars or head anchors to sell goods can make profit, because they need to pay high “pit fee” to occupy a “pit position” in the LS room of stars or head anchors while significantly reducing the LS sales price. Since the popularity of live streaming sales, internet celebrity anchors have charged brand retailers tens of thousands to hundreds of thousands of pit fees to promote products, but it is not uncommon for them to ultimately fail to achieve sales targets, bringing huge economic losses and rights protection costs to brand retailers. For example, in September 2020, a certain MCN (Multi-Channel Network) organization charged a "pit fee" of 200 thousand yuan to a retailer in Beijing, promising a guaranteed sales revenue of 250 thousand yuan, but the actual transaction was only 4 thousand yuan, the brand retailer sued the court and it took over 2 years to receive a refund; and in January 2022, an online company charged 70 thousand yuan to a retailer in Nanning, promising a guaranteed sales revenue of 1 million yuan, but only achieved a sales revenue of 278 yuan, the company’s request for a refund of the live streaming service fee was rejected. This data indicates significant conflicts of interest between brand retailers and anchors, which hinder their cooperation. Therefore, there is an urgent need to explore new cooperation models to deepen and strengthen the collaboration between brand retailers and anchors.

In order to create a more healthy and orderly LS service cooperation environment, the Ali V task settlement mode was upgraded in 2021, and the “equal proportion settlement” mode of pit fees was launched. The equal proportional settlement mode refers to the phased and tiered settlement of pit fees between brand retailers and anchors based on the actual sales ratio. If the anchor’s actual sales are less than 20% of the agreed sales, the platform will fully refund the pit fee to the brand retailer, and the anchor will not receive any payment. If the anchor’s actual sales reach or exceed 20% of the agreed sales, the platform will settle the pit fee proportionally: if 50% of the sales are achieved, 50% of the pit fee will be settled; if 70% of the sales are achieved, 70% of the pit fee will be settled. If sales exceed 100%, the pit fee will be settled as initially contract, and the brand retailer will not incur additional costs. For example, when the contract stipulates a sales volume of 100 items, the brand retailer needs to pay a pit fee of 1000 yuan to the anchor. If 19 items are actually sold, the pit fee is 0 yuan; 80 items were actually sold, with a pit fee of 800 yuan; If 100 or more items are actually sold, the pit fee is 1000 yuan.

This new settlement model is characterized by risk-sharing, incentives, and increased transparency and fairness. Firstly, brand retailers and anchors settle the pit fee based on the actual sales ratio, effectively sharing the risk between both parties and allowing brand retailers to avoid bearing the full cost if sales targets are not met. Secondly, this model incentivizes anchors to put more effort into promotions, as their income is directly tied to their sales performance, thereby enhancing overall sales effectiveness. Finally, the equal proportional settlement model enhances transparency and fairness in the settlement process, enabling brand retailers to clearly understand the basis for fee payments and reducing disputes arising from unmet sales expectations. This reform addresses the issue of high pit fees regardless of sales performance, providing greater security for brand retailers’ investments in live streaming marketing. By implementing an equal proportional settlement model for pit fees it protects the interests of brand retailers, extends the lifecycle and professionalism of anchors, and achieves a synergistic effect of 1+1>2 between anchors and brand retailers. For instance, the LS rooms of star anchors such as Lin Yilun and Li Jing have adopted this model, significantly increasing the ROI (sales revenue/time period expenses) for brand retailers, with each brand retailer saving an average of several tens of thousands of yuan in budget.

Therefore, how to optimize LS price discount and LS service effort decisions in live streaming commerce supply chain under the equal proportion settlement mode of “pit fee” has important theoretical value and practical guiding significance for supply chain members and the LS industry. In this paper, we consider a live streaming commerce supply chain system composed of a brand retailer and an Internet celebrity anchor and construct the pricing and live streaming service effort decision models under the equal proportion settlement mode of pit fee.

The main contributions of this paper are as follows. Firstly, we study the live streaming commerce supply chain composed of a brand retailer and an anchor and consider the decision of LS price discount rather than price, which can reflect the change of product price in the online shop and in LS room. Secondly, when assuming the market demand function in the LS room, we consider the cumulative effect of the anchor’s influence and LS effort on the purchase demand. Finally, we find the primary conditions for the establishment of the principal-agent partnership.

The remainder of this paper is organized as follows. Related studies are reviewed in Section 2, while the decision model is built and solved in Section 3. The numerical test is presented in Section 4. Finally, conclusions and future research directions are discussed in Section 5.

## 2. Literature review

This section sheds light on two streams of literature. The first stream addresses the influencing factors of watching live streaming and the impact of live streaming on consumers’ purchase intention, and the second stream addresses the live streaming commerce supply chain decisions.

The empirical research on live streaming mainly focuses on the influencing factors of customers watching live streaming and the impact of live streaming on consumers’ purchase intention. The main influencing factors are attitude, perceived value and viewing intention, viewing motivation [9], social interaction, community awareness, meeting new friends, entertainment, seeking information and external support lacking in real life [2], sex and humor appeals, social status display and interactivity [10], popularity of video content [11]. The influencing factors of customers’ purchase intention include atmosphere clue [12], Internet celebrities [13], mobile live video [14], contextual and environmental stimuli [15], information source characteristics [16], information process [17], relationship ties and customer commitment [18], price promotion, time pressure, interpersonal interaction, and visual appeal [19]. In addition, there ae some other empirical studies: motivation of the anchor to use the live streaming service [20], audience participation [21], social viewing strategy [22], IT affordance [7], operational strategy of Live Streaming Video companies [23], viewers social interaction in paid gifting [24], value co-creation behavior [25], mutual trust [26], impulse-buying [27], and so on.

Research on optimizing decision-making in the supply chain for live streaming commerce primarily falls into three main sub-streams: introduction strategies and mode selection for live streaming, pricing, and revenue distribution and incentive contracts.

In terms of the first sub-stream, Zhang and Tang [28] examined the impact of commission rates, fixed fees, and the influence of the anchor on manufacturers’ decisions to launch live streaming shopping channels. They found that the decision to open a live streaming shopping channel could either benefit or harm the manufacturer’s profit, depending on the interplay of these three factors. Considering the potential for consumer free-riding behavior, Huang et al. [29] explored the impacts and strategies of live stream channel introduction for competing retailers. They discovered that the equilibrium strategy regarding who introduces the live stream channel (i.e., no retailer introduces, one retailer introduces, and both retailers introduce) depends on the commission rate and mismatch cost. Zhang et al. [30] analyzed two modes (merchant live streaming and influencer live streaming) to determine retail prices and the level of promotional effort by streamers. Similarly, Yang et al. [31] investigated three live streaming sales modes selection considering consumer returns: e-commerce platform mode, transferring mode, and live streaming platform mode. Xin et al. [32] employed the Stackelberg game to illustrate three sales models: brand live streaming, influencer-led mixed live streaming, and influencer-led special live streaming. Wang et al. [33] considered a multi-echelon supply chain where an upstream supplier can sell products through a live streaming platform which can choose to sign either a reselling contract or an agency selling contract with the supplier.

In terms of live streaming pricing strategies, Mao et al. [34] conducted research on the pricing strategy for new products launched via live streaming, taking into account consumer uncertainty and network externalities. Ye et al. [35] developed a game model to analyze the seller’s preferences regarding influencer types (top and regular anchors) and pricing strategies (differential pricing strategy and uniform pricing strategy). They discovered that the seller’s preference for the type of anchor depends on the average bargaining power within the profession and the fixed payment required by top anchors. However, the aforementioned studies only address pricing in the context of live streaming commerce. In reality, consumers often receive discounts when purchasing products through a live room based on the brand pricing. Therefore, unlike the above studies, we consider the price discounts offered in live room. Regarding price discounts, Ji et al. [36] investigated the channel choice problem and price discount strategy within a live streaming e-commerce supply chain. Zhang et al. [37] designed incentive contracts considering the anchor’s influence and recommendation effort under information asymmetry, and investigated price discount decisions based on the linear contract cooperation model of "commission + pit fee". However, they did not consider the equal proportion settlement mode of the pit fee.

In terms of revenue distribution and incentive contract design, Wang et al. [38] analyzed the impact of the platform’s sharing rate on the compensation mechanism for the labor union and the anchor. Liu and Liu [5] studied the optimal decisions and coordination of live streaming sales under revenue-sharing contracts. These studies explored the joint decision-making process regarding income sharing and live streaming (LS) effort within the LS supply chain from different perspectives. Yang et al. [39] considered a scenario where the platform offers non-contracted and contracted streamers three different compensation plans, with streamers deciding their type and LS effort level. They found that by properly designing its compensation plans, the platform can screen out low-ability streamers and attract high-ability streamers to sign with it. In summary, most previous literature focused on empirical research on anchors, users, the interaction between anchors and users, and the joint decision-making regarding income sharing or pricing and LS effort. However, the joint optimal decision of LS price discount and LS service effort in the live e-commerce supply chain under the equal proportion settlement mode of the “pit fee” has not yet been studied. The research most closely related to our paper is Zhang and Xu [40], which discusses the proportional incentive contract based on target sales volume and studies contract design optimization using principal-agent theory. However, their study did not consider price discount decisions.

Compared to existing literature, despite the growing popularity of live streaming commerce, there is a notable lack of research on joint pricing and service effort strategies within the live streaming commerce supply chain under the equal proportion settlement mode. Therefore, distinct from the aforementioned studies, our research focuses on this new cooperation mode to deepen the collaborative relationship between brand retailers and anchors. Furthermore, within this mode, our study examines the brand retailer’s price discount strategies and the anchor’s service effort strategies, while also considering the impact of the anchor’s influence on optimal decisions and profits within the live streaming commerce supply chain. By exploring these aspects, our paper highlights the similarities and differences between our research and related studies from four perspectives: price discounts, anchor’s influence, anchor’s service efforts, and the equal proportion settlement mode, aiming to address the gaps in existing literature. As shown in Table 1, previous literature has studied certain aspects in isolation, whereas our paper comprehensively explores all these crucial topics in the context of live streaming commerce.

**Table 1:** Positioning of this study in the literature.

Papers	Price discount	Service effort	Influence of the anchor	Equal proportion settlement mode
Zhang and Tang [28]; Zhang et al. [41]; Zhang et al. [30]			√	
Xin et al. [32]; Wang et al. [38]; Yang et al. [39]		√		
Zhang et al. [42]		√	√	
Ji et al. [36]; Wan et al. [43]	√	√		
Zhang et al. [37]	√	√	√	
Zhang and Xu [40]		√	√	√
Our paper	√	√	√	√

## 3. Problem description and assumptions

The live streaming e-commerce supply chain under the equal proportion settlement mode of pit fee consists of a brand retailer who opens an online shop on an e-commerce platform and a celebrity anchor who signs a contract with an MCN live streaming organization studied in this paper.

**Fig 1:**
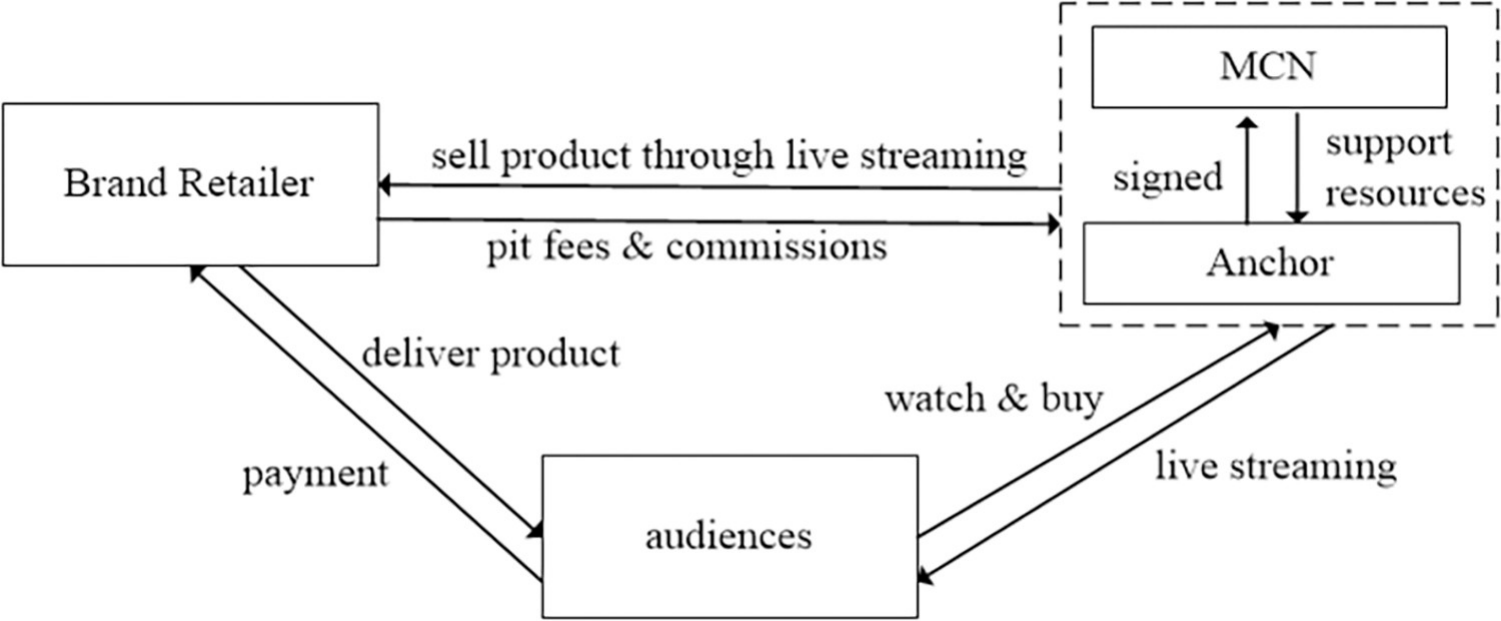
The live streaming e-commerce supply chain.

The anchor is a provider of LS content, showcasing and recommending products to audiences through the LS platform. The anchor needs to possess certain professional knowledge and good communication skills in order to attract audiences and facilitate transactions. The MCN organization is the anchor’s broker and is responsible for the management, training, and resource coordination of the anchor. The MCN organization provides product resources, technical support, and brand promotion support to the anchor through cooperation with the brand retailer, helping the anchor improve the effectiveness and influence of live streaming. The brand retailer is the provider of products, showcasing and selling products to potential consumers through live streaming in collaboration with the anchor and MCN organization. The brand retailer needs to provide competitive products and reasonable prices to attract audiences to purchase. The relationship between these three is interdependent and mutually beneficial. The anchor brings sales to the brand retailer through live streaming while obtaining pit fees and commissions from the brand retailer; the MCN, on the other hand, provides support and resources for the anchor to improve his live streaming performance and attract more brand retailers to cooperate; the brand retailer expands brand exposure and sales through live streaming to achieve marketing goals. The relationships between various roles in the live streaming e-commerce supply chain are shown in Fig 1.

In the live streaming e-commerce supply chain, the brand retailer is the principal and the anchor is the agent, the brand retailer needs to pay the pit fee and sales commission to the anchor. Taking Taobao Live as an example, the distribution mechanism of sales commissions (including pit fees) is carried out in a ratio of 1:2:7, with 10% belonging to Alibaba, 20% belonging to the Taobao Live platform, and 70% paid to MCN organization. The MCN organization then distributes the commission to the contracted anchor according to the negotiated ratio, such as 8:2, 7:3, 5:5, or 4:6. In our study, we only consider the revenue distribution between the brand retailer and the anchor, without taking into account the revenue distribution between the MCN and the anchor. Thus, the MCN and the anchor are regarded as a single entity in our study.

The notations used in the modeling and their definitions are included in Table 2.

**Table 2:** Notations and definitions.

Notation	Definition
*δ*	Price discount in the LS room (decision variable, 0<*δ*<1)
*e*	live streaming service effort of the anchor (decision variable)
*p*	Price of the product
*c*	Unit product cost of the brand retailer
*k*	Sales commission rate
*a*	Potential market demand in the LS room
*b*	Consumers’ price sensitivity in LS room
*h*	Cost coefficient of live streaming service effort
*η*	Effect coefficient of live streaming service effort
*i*	The influence of the anchor
*λ*	Sensitivity of consumers to the anchor’s LS marketing (0<*λ*<*b*)
*α*	Proportion of LS service platform extracted from the anchor commission (0<*α*<1)
*β*	Sales commission rate paid by the brand retailer to the e-commerce platform (0<*β*<1)
*L*	Pit fee
*T*	Agreed sales volume
*ε*	The threshold of the ratio of paid-successfully sales volume to agreed sales volume
*r*	The ratio of confirmed-receipt sales volume to paid-successfully sales volume (0<*ε*<*r*<1), i.e., the retention rate of consumers
*D*	Consumer demand in the LS room
Π_1_,Π_2_	Total profit of the head anchor and the brand retailer

**Fig 2:**
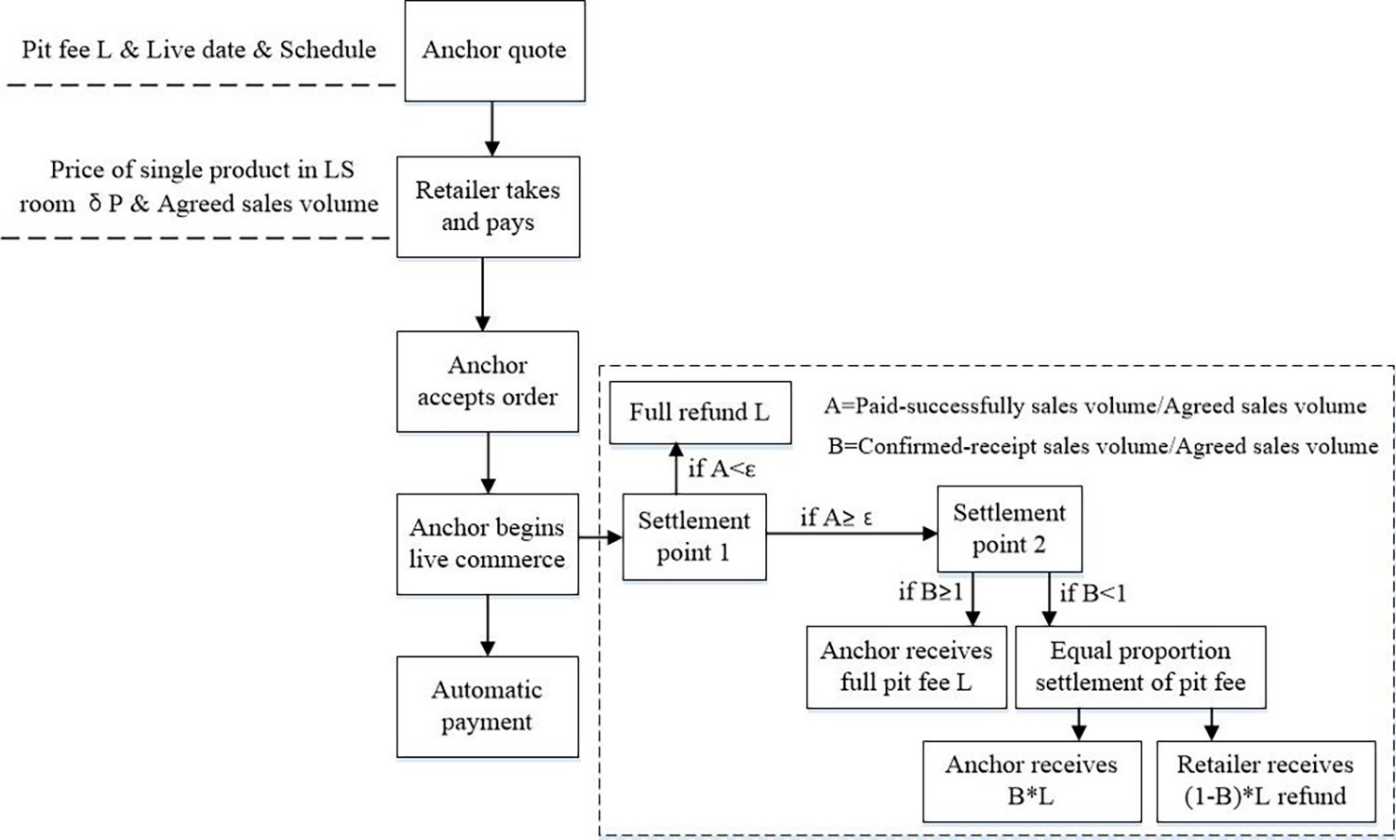
The process of equal proportional settlement mode of pit fee.

The equal proportion settlement mode of the pit fee is an upgrade of the one-price mode of the pit fee, and the income composition of the anchor consists of commissions and fixed pit fees. The process of equal proportional settlement mode of pit fee is shown in Fig 2.

As shown in Fig 2, brand retailers can recruit anchors on the Ali V Task Platform and set parameters such as agreed sales *T*and price discounts *δ*. After the brand retailer reaches a cooperation agreement with the anchor, the anchor will promote the brand retailer’s products at the agreed time. After the anchor starts live streaming, in the first settlement point, such as within 15 days after the live streaming, if the ratio of paid-successfully sales volume to agreed sales volume is less than a threshold *ε*, the pit fee will be refunded in full. If the ratio of paid-successfully sales volume to agreed sales volume is more than the threshold *ε*, the task will be advanced to the second settlement point, such as within 30 days after the live-streaming. On the 30th day after the live-streaming, the platform will calculate the ratio of confirmed-receipt sales volume to agreed sales volume and settle the pit fee to the anchor in equal proportion. If the ratio of confirmed-receipt sales volume to agreed sales volume is more than 1, the brand retailer does not need to pay more pit fees to the anchor, just pay a fixed pit fee *L*. In this case, brand retailers can also give additional rewards to the anchor. Since the pit fee and the sales commission rate are relatively fixed numbers, which are not affected by the paid-successfully sales volume, we do not consider the decision of the pit fee *L* and sales commission rate *k* in this paper but only consider the joint decision of the price discount *δ* and the anchor’s live streaming service effort *e*.

Based on the above description, in order to simplify the game decision model of equal proportional settlement mode of pit fee, combined with the live streaming commerce operation practice, we make the following basic assumptions:

**(1) The impact of anchor marketing on demand:** In the equal proportional settlement mode of pit fee, the income of the anchor is completely related to the anchor’s performance. Therefore, the anchor can only increase his income by increasing his LS service efforts. The anchor’s LS service effort *e* refers to the efforts made by the anchor to ensure the effect of live streaming, such as efforts to improve the anchor’s own ability, the anchor’s control over product quality and familiarity with product function selling points, the anchor’s interaction with consumers and the incentive to followers in the LS room, and the short videos produced and released by the anchor for publicity and promotion on various new media platforms for content grass planting and LS preheating, etc. In addition, the anchor’s income also depends on the anchor’s influence. The greater the influence of the anchor, the more his fans, and the greater his pull-on demand. In summary, the anchor’s influence *i* and LS service effort *e* can increase demand *λ*(*i*+*ηe*), where *η* is the effect coefficient of LS service effort and *λ* is the sensitivity of consumers to the anchor’s LS marketing [21].

**(2) Consumer demand:** Consumers always think that the product price in the LS room will be lower because they believe that the anchor can get a good discount *δ* from the brand retailer; that is, consumers pay *δp* to buy the product. According to assumption (1), consumer demand is mainly influenced by price, anchor marketing, and market potential demand. Therefore, we assume the consumer demand or paid-successfully sales volume in the LS room is:

D=a−bδp+λ(i+ηe).

Where the parameter *a* represents the potential market demand in the LS room and the parameter *b* represents the consumers’ price sensitivity in the LS room. We assume that within the same market segment, consumer price sensitivity *b* is relatively consistent. This is because, on the same live streaming platform and with similar product categories, consumer behavior and price response tend to exhibit a high degree of consistency.

**(3) Cost structure:** We assume that the unit product cost of the brand retailer is *c*. The operating costs of intermediary platforms such as Ali V Task are negligible. The anchor needs to incur certain costs when making efforts in live broadcast services. Based on the assumptions of costs in existing studies (such as Gao et al., [44]), it is assumed that the anchor’s service effort cost satisfies a quadratic function, which is *he*^2^/2, where *h* is the cost coefficient of live streaming service effort.

**(4) Parameter Relationship:** Firstly, based on the operational practice of live streaming commerce, we assume that the threshold of the ratio of paid-successfully sales volume to agreed sales volume is satisfies 0<*ε*<0.5. For example, the Taobao platform sets this threshold to 0.2. Secondly, assume that the retention rate of consumers (i.e., ratio of confirmed-receipt sales volume to paid-successfully sales volume) *r* satisfies 0.5<*r*<1. This is because in the live streaming commerce industry, the consumer return rate generally does not exceed 50%, so the retention rate of consumers is greater than 50%. Obviously, there is 0<*ε*<*r*<1. In addition, consumers rush to buy in the LS room, often focusing on the preferential price and secondly on the anchor’s marketing. Therefore, we assume that consumers are more sensitive to prices than to anchor marketing, that is 0<*λ*<*b*. Finally, it is clear that the commission rates of anchors and e-commerce platforms are satisfied 1−*k*−*β*>0.

## 4. Model construction and solution

In this section, we build the profit models of both sides under the equal proportion settlement mode of “pit fee”, and then solve the models.

Obviously, the revenue of the star or head anchor includes the pit fee and the sales commission, and the expenditure is the sales commissions drawn by the LS service platform and the cost of the LS effort. The cost of LS effort mainly includes the cost of live advertising placement, the cost of purchasing traffic, the cost of manpower and time invested by the live streaming team, the cost of sending red envelopes or lottery draws in the live streaming room, and so on. The revenue of the brand retailer is the sales income after deducting the product cost, and the expenditure includes the pit fee, the sales commission paid to the anchor, and the technical service fee paid to the e-commerce platform.

After the cooperation agreement is reached, the anchor team will make LS efforts to sell the product through live streaming. Therefore, the supply chain under the equal proportion settlement model of “pit fee” is a brand retailer-led and constrained LS commerce supply chain, and the order of decision-making is as follows: first, the brand retailer decides the price discount of the product in the LS room *δ*, and then the anchor decides the LS service effort *e*.

If the ratio of paid-successfully sales volume to agreed sales volume is less than *ε*, i.e., *D*/*T*<*ε*, the pit fee will be refunded in full. That is, if *D*<*εT*, the retailer does not have to pay the high pit fee *L* = 0. Thus, we obtain the decision optimization problem as follows:

$$\left\{ \begin{array}{l} \max\Pi_{1}(e)=k\delta pr(1-\alpha )(a-b\delta p+\lambda (i+\eta e))-he^{2}/2\\ \max\Pi_{2}(\delta )=r((1-k-\beta )\delta p-c)(a-b\delta p+\lambda (i+\eta e))\\ s.t.\;a-b\delta p+\lambda (i+\eta e)<\varepsilon T \end{array}\right.$$ (1)

By solving the constrained bi-objective optimization problem (1), we obtain the following Proposition 1.

**Proposition 1.** If the ratio of paid-successfully sales volume to agreed sales volume is less than *ε*, the retailer’s optimal product price discount and the anchor’s optimal LS effort are $\delta^{*}=\frac{hA+cB}{2pB(1-k-\beta )}$ and $e^{*}=\frac{kr\lambda \eta (1-\alpha )(hA+cB)}{2hB(1-k-\beta )}$, respectively, where *A* = (*a*+*iλ*)(1−*k*−*β*), *B* = *bh*−(1−*α*)*krη*^2^*λ*^2^. Furthermore, the optimal demand or sales in the LS room is $D^{*}=\frac{hA-cB}{2h(1-k-\beta )}$, the anchor’s maximum profit is $\Pi_{1}^{*}=\frac{kr(1-\alpha )(hA+cB)(hA(3B-bh)-cB(B+bh))}{8h{\left(1-k-\beta \right)}^{2}B^{2}}$, the brand retailer’s maximum profit is $\Pi_{2}^{*}=\frac{r{\left(hA-cB\right)}^{2}}{4hB(1-k-\beta )}$.

Please refer to S1 Appendix for the proofs of Proposition 1.

Proposition 1 shows that when the paid-successfully sales volume is low, the optimal decisions of brand retailers and anchors exist, but they are not related to the threshold of the ratio of paid-successfully sales volume to agreed sales volume, nor to agreed sales volume, and the constraints in the LS e-commerce supply chain decision-making model become invalid. Obviously, the full refund of the pit fee settlement mode provides protection for retailers and also demonstrates the weak live commerce ability of anchors.

Based on the constraint of problem (1) and the optimal decision of Proposition 1, we can derive the following Lemma 1.

**Lemma 1.** When the ratio of paid-successfully sales volume to agreed sales volume is less than *ε*, the feasible condition for the optimal decision is $i<\frac{cB-(1-k-\beta )(a-2\varepsilon T)h}{(1-k-\beta )\lambda h}$.

Please refer to S1 Appendix for proof of Lemma 1.

Lemma 1 indicates that if the influence of the anchor is minimal, leading to a small number of fans and consequently a low number of audiences entering the anchor’s LS room, the product sales will be lower. Therefore, the paid-successful sales volume cannot reach the agreed sales volume, and the pit fee will be 0.

If the ratio of paid-successfully sales volume to agreed sales volume is greater than *ε*, and the ratio of confirmed-receipt sales volume to agreed sales volume is less than 1, i.e., *D*/*T*≥*ε* & *rD*/*T*<1, the LS service platform will settle the pit fee to the anchor in equal proportion. That is, if *εT*≤*D*<*T*/*r*, the pit fee paid by the brand retailer to the anchor is *rDL*/*T*. Thus, we obtain the decision optimization problem as follows:

$$\left\{ \begin{array}{l} \max\Pi_{1}(e)=r((1-\alpha )k\delta p+L/T)(a-b\delta p+\lambda (i+\eta e))-he^{2}/2\\ \max\Pi_{2}(\delta )=r((1-k-\beta )\delta p-c-L/T)(a-b\delta p+\lambda (i+\eta e))\\ s.t.\;\varepsilon T\leq a-b\delta p+\lambda (i+\eta e)<T/r \end{array}\right.$$ (2)

By solving the constrained bi-objective optimization problem (2), we obtain the following Proposition 2.

**Proposition 2.** If the ratio of paid-successfully sales volume to agreed sales volume is greater than *ε*, and the ratio of confirmed-receipt sales volume to agreed sales volume is less than 1, the optimal price discount is $\delta^{*}=\frac{(hA+cB)T+(2B-bh)L+E}{2pBT(1-k-\beta )}$, and the optimal LS effort is $e^{*}=\frac{r\eta \lambda (F+G-H)}{2hBT(1-k-\beta )}$, where *E* = *rLη*^2^*λ*^2^ (1−*β*−*αk*), *F* = *bhL*(2−2*β*−*k*−*αk*), *G* = *kT*(1−*α*)(*hA*+*cB*), *H* = *L*(1−*β*−*αk*)(*bh*−*B*). Furthermore, the optimal demand or sales in the LS room is $D^{*}=\frac{T(hA-cB)-bhL+E}{2hT(1-k-\beta )}$, the anchor’s maximum profit is $\Pi_{1}^{*}=\frac{r(H-F-G)[h(bL+bcT-TA)(3B-bh)+r\eta^{2}\lambda^{2}H+cT{\left(bh-B\right)}^{2}]}{8hT^{2}B^{2}{\left(1-k-\beta \right)}^{2}}$, and the retailer’s maximum profit is $\Pi_{2}^{*}=\frac{r{\left[T(hA-cB)-bhL+E\right]}^{2}}{4hBT^{2}(1-k-\beta )}$.

Please refer to S1 Appendix for the proofs of the Proposition 2.

Proposition 2 reveals that when the paid-successfully sales volume is high, the optimal decisions of brand retailers and anchors exist, and the pit fee has a positive promoting effect on the optimal decisions. In this equal proportional settlement mode of pit fee, anchors will invest more LS efforts in order to obtain a higher proportion of pit fee, and brand retailers will also offer higher price discounts to attract consumers to purchase in order to obtain higher sales revenue. Under the dual effect, the product has a higher cost performance, and the number of customers confirm receipt also increases, resulting in greater profits for both anchors and brand retailers.

Based on the constraint of the problem (2) and the optimal decision of Proposition 2, we can get the following Lemma 2.

**Lemma 2.** When the ratio of paid-successfully sales volume to agreed sales volume is greater than *ε*, and the ratio of confirmed-receipt sales volume to agreed sales volume is less than 1, the feasible condition for the optimal decision is $\frac{bhL+cBT-E-(1-k-\beta )(a-2\varepsilon T)hT}{(1-k-\beta )\lambda hT}\leq i<\frac{r(bhL+cBT-E)-(1-k-\beta )(ar-2T)hT}{(1-k-\beta )\lambda hrT}$.

Please refer to S1 Appendix for the proofs of the Lemma 2.

Lemma 2 states that if the influence of the anchor reaches a certain level, the payment conversion rate in the LS room will be higher than a specific value, and if the confirmed-receipt sales volume is not high enough, the anchor can only receive an equal proportion of the pit fee, not the full amount.

If the ratio of confirmed-receipt sales volume to agreed sales volume is greater than or equal to 1, that is, if *D*≥*T*/*r*, the brand retailer needs to pay full pit fee *L* to the anchor. Thus, we obtain the decision optimization problem as follows:

$$\left\{ \begin{array}{l} \max\Pi_{1}(e)=(1-\alpha )kr\delta p(a-b\delta p+\lambda (i+\eta e))-he^{2}/2+L\\ \max\Pi_{2}(\delta )=r((1-k-\beta )\delta p-c)(a-b\delta p+\lambda (i+\eta e))-L\\ s.t.\;a-b\delta p+\lambda (i+\eta e)\geq T/r \end{array}\right.$$ (3)

By solving the constrained bi-objective optimization problem (3), we obtain the following Proposition 3.

**Proposition 3.** If the ratio of confirmed-receipt sales volume to agreed sales volume is greater than or equal to 1, the optimal price discount is $\delta^{*}=\frac{hI}{prB}$, and the optimal LS effort is $e^{*}=\frac{(1-\alpha )k\eta \lambda I}{B}$, where *I* = *r*(*a*+*iλ*)−*T*. Furthermore, the optimal demand or sales in the LS room is $D^{*}=\frac{T}{r}$, the anchor’s maximum profit is $\Pi_{1}^{*}=\frac{2rLB^{2}+hkI(1-\alpha )(2BT-bhI+IB)}{2rB^{2}}$, and the brand retailer’s maximum profit is $\Pi_{2}^{*}=\frac{hT[T(1-\beta -k)-rA]-rB(L+cT)}{rB}$.

Please refer to S1 Appendix for the proofs of the Proposition 3.

Proposition 3 shows that when the ratio of confirmed-receipt sales volume to agreed sales volume is greater than or equal to 1, the optimal decisions of brand retailers and anchors exist, and the optimal demand is only affected by the ratio of confirmed-receipt sales volume to paid-successfully sales volume. The larger the ratio of confirmed-receipt sales volume to paid-successful sales volume, the higher the confirmed-receipt sales volume, and the less the returned sales without reason. This indicates that the higher the cost performance or quality of the product, the easier it is to achieve the agreed sales volume. However, as the pit fee has already been capped, the stimulation for the anchor is not significant enough. Therefore, it is possible to consider adding agreement terms to provide additional rewards for the anchor’s overfulfilled sales.

Based on the constraint of problem (3) and the optimal decision of Proposition 3, the following Lemma 3 can be obtained.

**Lemma 3.** When the ratio of confirmed-receipt sales volume to agreed sales volume is greater than or equal to 1, the feasible condition for the optimal decision is $i>\frac{T-ra}{\lambda r}$.

Please refer to S1 Appendix for proof of Lemma 3.

Lemma 3 reflects that if the influence of the anchor exceeds a certain threshold, there exists an optimal supply chain decision that allows the anchor to receive a full pit fee.

Therefore, the optimal decision, optimal demand, and maximum profit under the equal proportional settlement mode of pit fee are shown in Table 3.

**Table 3:** The optimal values under the equal proportional settlement mode of pit fee.

Scenario	δ*	e*	D*	$\Pi_{1}^{*}$	$\Pi_{2}^{*}$
*D*/*T*<*ε*	$\frac{hA+cB}{2pB(1-k-\beta )}$	$\frac{kr\lambda \eta (1-\alpha )(hA+cB)}{2hB(1-k-\beta )}$	$\frac{hA-cB}{2h(1-k-\beta )}$	$\frac{kr(1-\alpha )(hA+cB)[hA(3B-bh)-cB(B+bh)]}{8h{\left(1-k-\beta \right)}^{2}B^{2}}$	$\frac{r{\left(hA-cB\right)}^{2}}{4hB(1-k-\beta )}$
*D*/*T*≥*ε* & *rD*/*T*<1	$\frac{(hA+cB)T+(2B-bh)L+E}{2pBT(1-k-\beta )}$	$\frac{r\eta \lambda (F+G-H)}{2hBT(1-k-\beta )}$	$\frac{T(hA-cB)-bhL+E}{2hT(1-k-\beta )}$	$\frac{r(H-F-G)[h(bL+bcT-TA)(3B-bh)+r\eta^{2}\lambda^{2}H+cT{\left(bh-B\right)}^{2}]}{8hT^{2}B^{2}{\left(1-k-\beta \right)}^{2}}$	$\frac{r{\left[T(hA-cB)-bhL+E\right]}^{2}}{4hBT^{2}(1-k-\beta )}$
*rD*/*T*≥1	$\frac{hI}{prB}$	$\frac{(1-\alpha )k\eta \lambda I}{B}$	$\frac{T}{r}$	$\frac{2rLB^{2}+hkI(1-\alpha )(2BT-bhI+IB)}{2rB^{2}}$	$\frac{hT[T(1-\beta -k)-rA]-rB(L+cT)}{rB}$

In addition, according to the first-order conditions and the envelope theorem, we obtain the following **Corollary 1**.

**Corollary 1.** In the live streaming commerce supply chain with equal proportion settlement mode of pit fee, both δ* and e* increase in *λ*, *i*, *η*, and *r*, and decrease in *h*.

### Please refer to S1 Appendix for the proofs of Corollary 1

Corollary 1 indicates that the optimal price discount and the optimal LS service effort increase with the increase of the sensitivity of consumers to the anchor’s LS marketing, the influence of the anchor, the effect coefficient of LS effort, and the ratio of confirmed-receipt sales volume to paid-successfully sales volume, while decrease with the decrease of the unit cost of LS effort.

In fact, the stronger the sensitivity of consumers to the anchor’s LS marketing, the greater the influence of the anchor, the greater the effect coefficient of LS effort, and the higher the ratio of confirmed-receipt sales volume to paid-successfully sales volume, the greater the demand for LS rooms, and the stronger the willingness of users to purchase. At this time, anchors are willing to invest more in LS service efforts to sell products, and brand retailers are also willing to offer greater price discounts to promote product sales so that both parties can obtain greater profits.

In addition, price discounts and LS service efforts decrease as the cost coefficient of LS service efforts increases. The fundamental reason is that the rising cost of efforts impacts the maximization of economic benefits. When h increases, the cost per unit of effort rises, making investments in improving streaming quality or increasing interactions more expensive. To control total costs and avoid scenarios where revenues cannot cover costs, anchors tend to reduce their efforts. Additionally, as effort costs rise, the marginal cost of increasing LS service efforts also increases. When the marginal cost exceeds the marginal benefit, further increasing efforts becomes uneconomical. To maximize net profits, the optimal strategy is to reduce effort input. The size of price discounts is closely related to the anchor’s effort and the effectiveness of product promotion. Higher effort costs reduce the anchor’s promotional enthusiasm, thereby affecting the price discount strategy. When efforts are reduced, the attractiveness of price discounts may be insufficient to draw more consumers, leading brand retailers to adjust price discounts to balance costs and expected revenues. Therefore, as the cost coefficient of LS service efforts increases, both price discounts and LS service efforts decrease to ensure the maximization of economic benefits.

## 5. Numerical analysis

In this section, we first verify the impact of key parameters on the optimal LS service effort of the anchor, optimal price discount in the LS room, optimal demand, and the optimal total profit of the anchor and the brand retailer.

According to Propositions 1, 2, and 3 in Section 3, and in combination with the e-commerce LS industry practice, the data setting for the base parameters in the numerical analysis is as follows: *p* = 300; *c* = 50; *k* = 0.2; *a* = 2000,3000,10000;*b* = 8, *λ* = 1; α=0.2;β=0.05;L=200000;T=5000;ε=0.2;r=0.8;i=25;h=1.

Given the numerous parameters involved in this study, we have chosen to conduct a sensitivity analysis on the key parameters that significantly impact the equal proportion settlement mode. In practice, since e-commerce platforms typically charge a fixed commission of 5%, we exclude the commission paid by the brand retailer to the e-commerce platform from our analysis and focus only on the interaction factors between the brand retailer and the anchor. Additionally, product price and cost are generally confirmed by the brand retailer before cooperation, so these factors are also excluded from the sensitivity analysis. Similarly, the commission parameters between the MCN organization and the anchor are not considered. Ultimately, we selected the key *h*, *r*, *i* and *k* to examine their effects on the optimal decisions, demand, and profits of both the brand retailer and the anchor.

**Fig 3:**
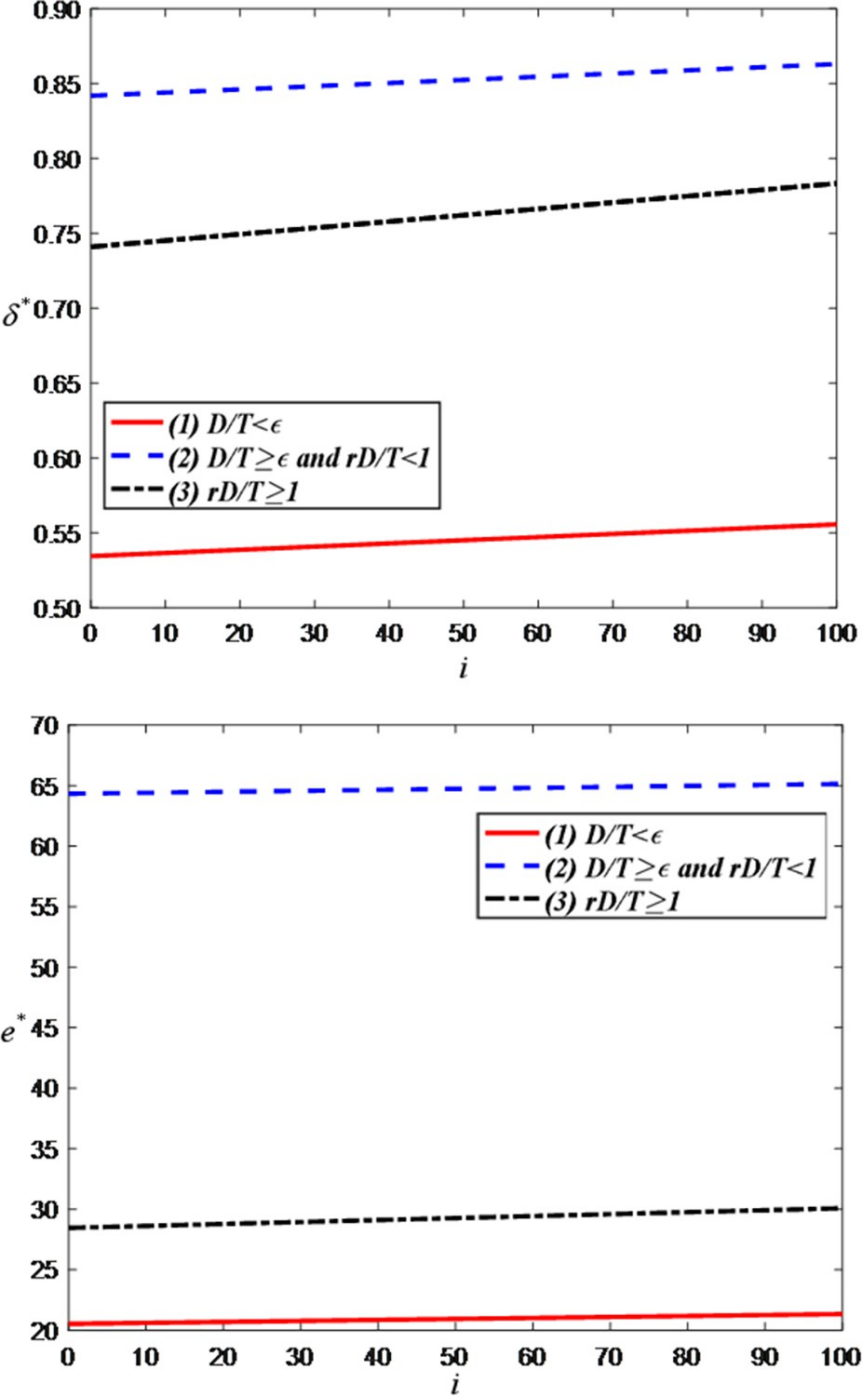
The impact of the influence of the anchor on optimal decisions. (a) price discount of the brand retailer. (b) LS service effort of the anchor.

Firstly, we examine the impact of the influence of the anchor on the optimal decisions and maximum profits and set the value range of *i* as [0, 100]. Fig 3(A) illustrates that the greater the influence of the anchor, the larger the price discount in the LS room. This is because as the influence of the anchor grows, so does the size of their audience. When an anchor has a larger fan and audience base, brand retailers tend to attract more viewers by offering greater price discounts, thereby increasing advertising exposure and sales volume. Fig 3(B) indicates that the greater the influence of the anchor, the more attention and expectations they receive. As the number of fans increases, the anchor’s live streaming service effort directly impacts audience satisfaction and loyalty. Therefore, to maintain and enhance their influence, anchors typically increase their investment in live streaming service, including improving livestream quality, enhancing content innovation, strengthening interaction with the audience, etc., to meet the demands of fans and maintain competitiveness in the fiercely competitive live streaming industry.

**Fig 4:**
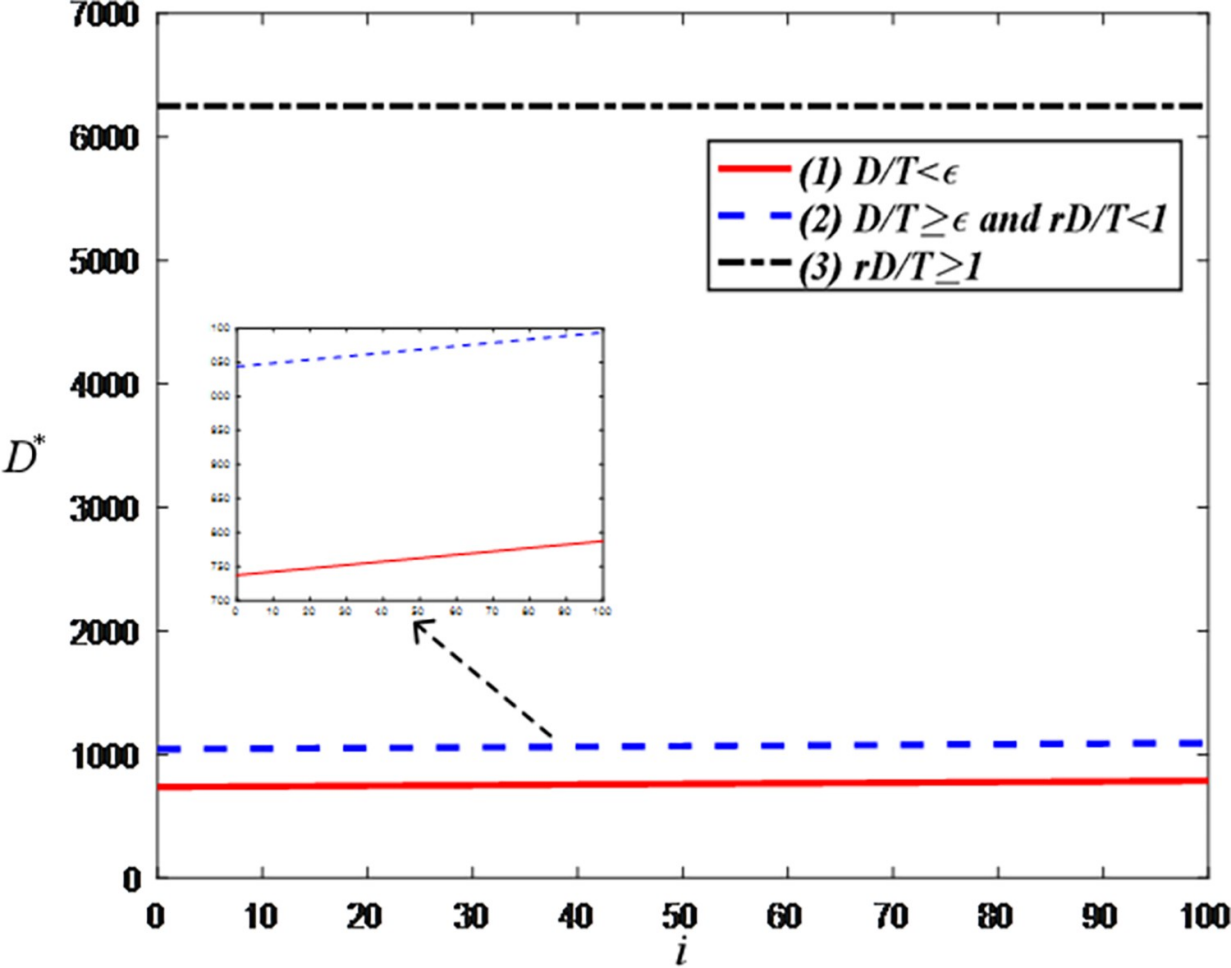
The impact of the influence of the anchor on optimal demand.

Fig 4 indicates that the greater the anchor’s influence, the higher the consumer demand. This is evident because high-influence anchors can increase product exposure and consumers’ willingness to purchase. However, when demand reaches a certain threshold, the optimal demand becomes independent of the anchor’s influence. This is because, under the equal proportion settlement mode, further increases in demand do not significantly enhance the anchor’s revenue. Hence, the anchor will not increase its influence to improve demand.

**Fig 5:**
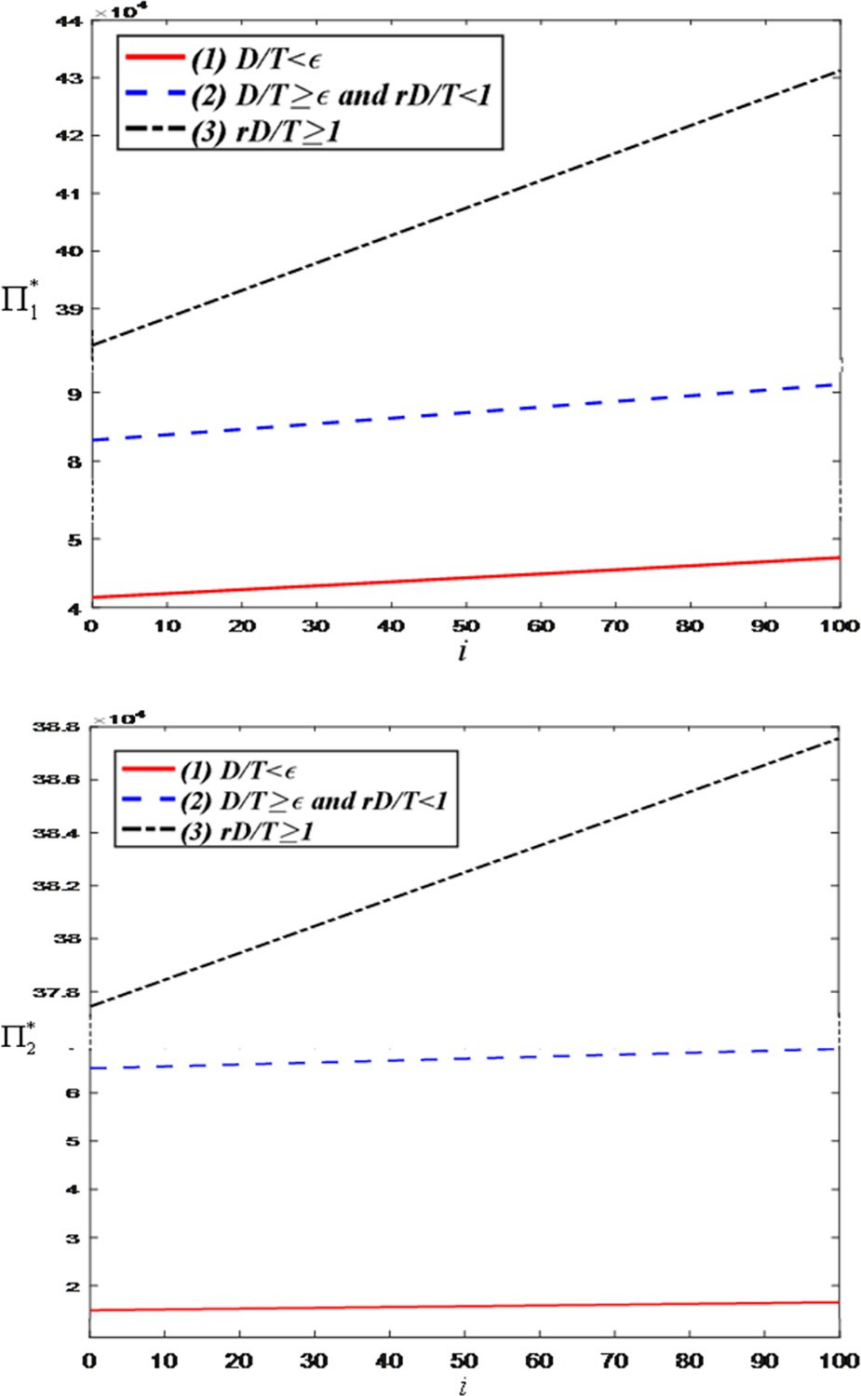
The impact of the influence of the anchor on total profit of the head anchor and the brand retailer. (a) Total profit of the head anchor. (b) Total profit of the brand retailer.

Fig 5(A) and 5(B) demonstrates that the greater the influence of the anchor, the higher the profits for both the anchor and the brand retailer. This is attributed to the advertising effect generated by the anchor’s influence in the live streaming commerce model. As the anchor’s influence grows, the number of viewers and attention attracted to their livestreams increases, resulting in greater exposure of brand retailers’ products within the anchor’s LS room. This increased exposure and influence attract more brand retailers willing to collaborate with the anchor, thereby enhancing the anchor’s overall profits. Additionally, since the anchor’s influence can drive consumer purchasing intentions, there is potential for an increase in the sales volume of brand retailers’ products, further boosting their profits.

**Fig 6:**
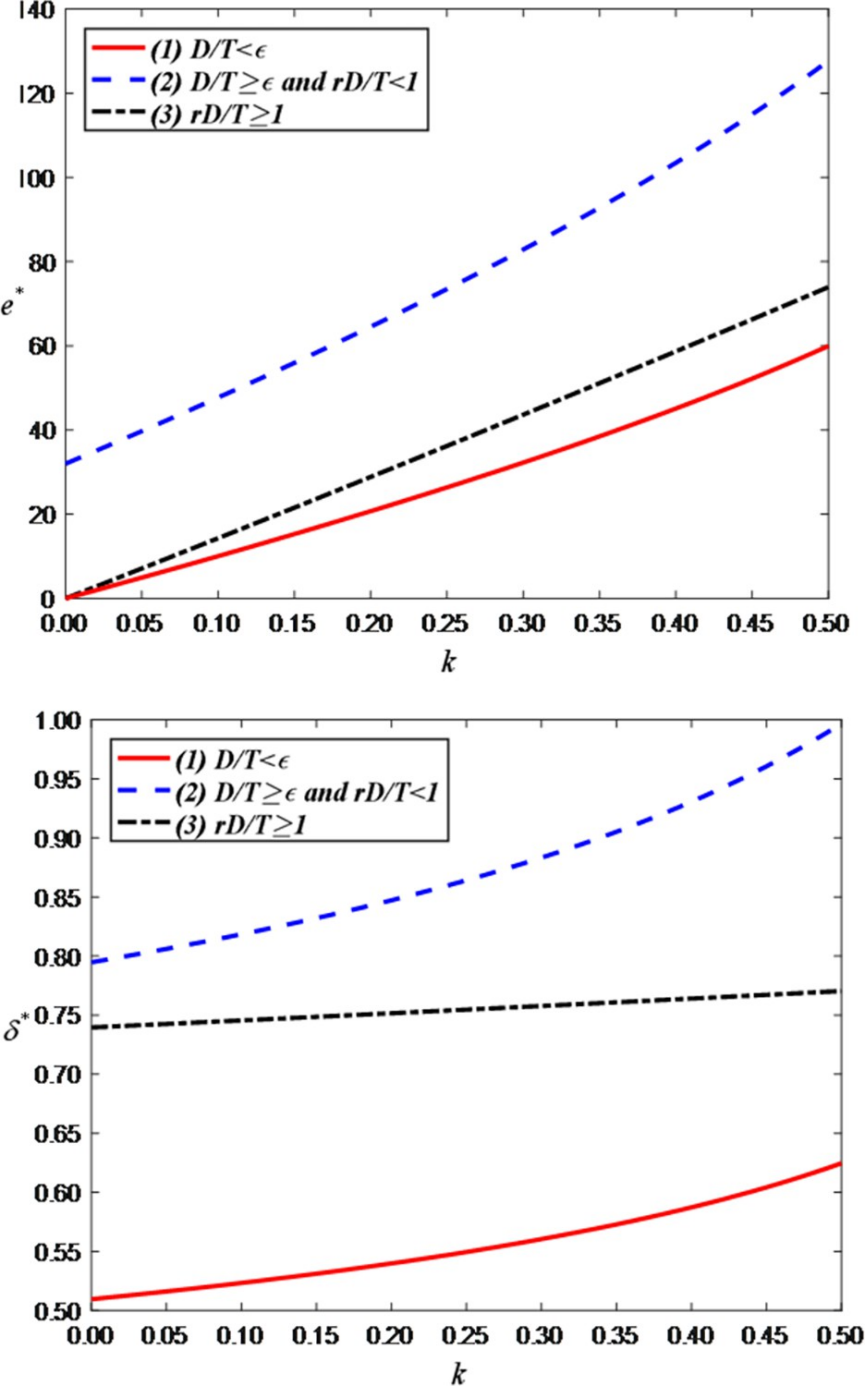
The impact of cost coefficient of LS service effort on optimal decisions. (a) LS service effort of the anchor. (b) Price discount.

Secondly, we explore the impact of the cost coefficient of LS service effort on the optimal decisions and maximum profits and set the value range of *h* as [0.5, 1]. Fig 6(A) illustrates that as the cost coefficient of LS service effort increases, the live streaming service effort of the anchor decreases. This is because the increase in costs may impose economic pressure and resource constraints on the anchor. If the anchor needs to spend more time, energy, and funds to improve the quality of live streaming services, but the income does not correspondingly increase, they may face financial difficulties. This could lead to a reduction in the anchor’s investment in livestreaming services, such as reducing content innovation, lowering interaction frequency, or providing simpler programs to reduce costs and maintain economic balance. Therefore, the increase in the unit cost of the live streaming service effort may indirectly lead to a decrease in the live streaming service effort of the anchor. Fig 6(B) indicates that as the cost coefficient of live streaming service effort increases, the price discount in the LS room decreases. This is because an increase in the effort to provide live streaming services can result in a reduction in the anchor’s profit margin. If the anchor incurs additional costs to enhance the live streaming service effort, brand retailers may reduce the price discount to maintain profitability. This implies a need for brand retailers to balance costs and profits, and as costs increase, price discounts may decrease accordingly to sustain a viable profit model.

**Fig 7:**
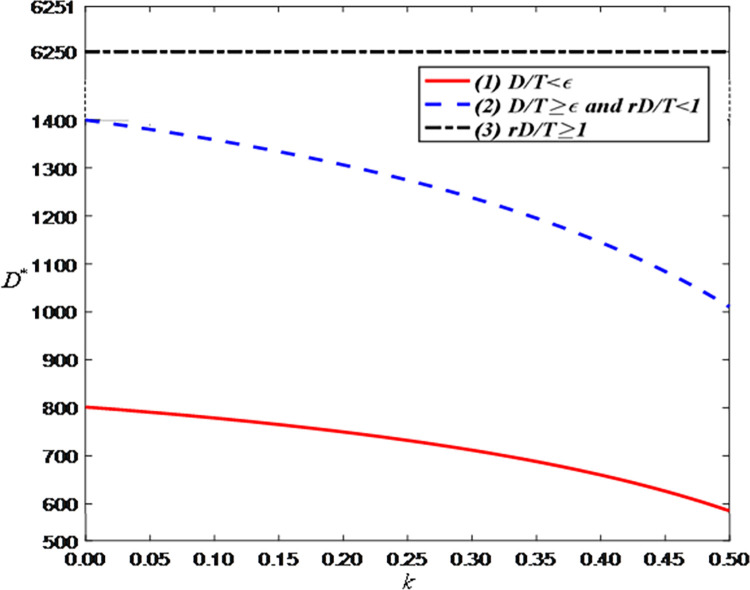
The impact of cost coefficient of LS service effort on optimal demand.

Fig 7 shows that when the demand is less than *T*/*r*, the optimal demand decreases as the LS service effort cost coefficient increases. This is because, as the effort cost increases, the anchor’s willingness to make efforts diminishes, thereby reducing demand. On the contrary, when the demand is greater than the threshold, the optimal demand is related to the retention rate of consumers and the agreed sales volume but has nothing to do with the effort cost coefficient.

**Fig 8:**
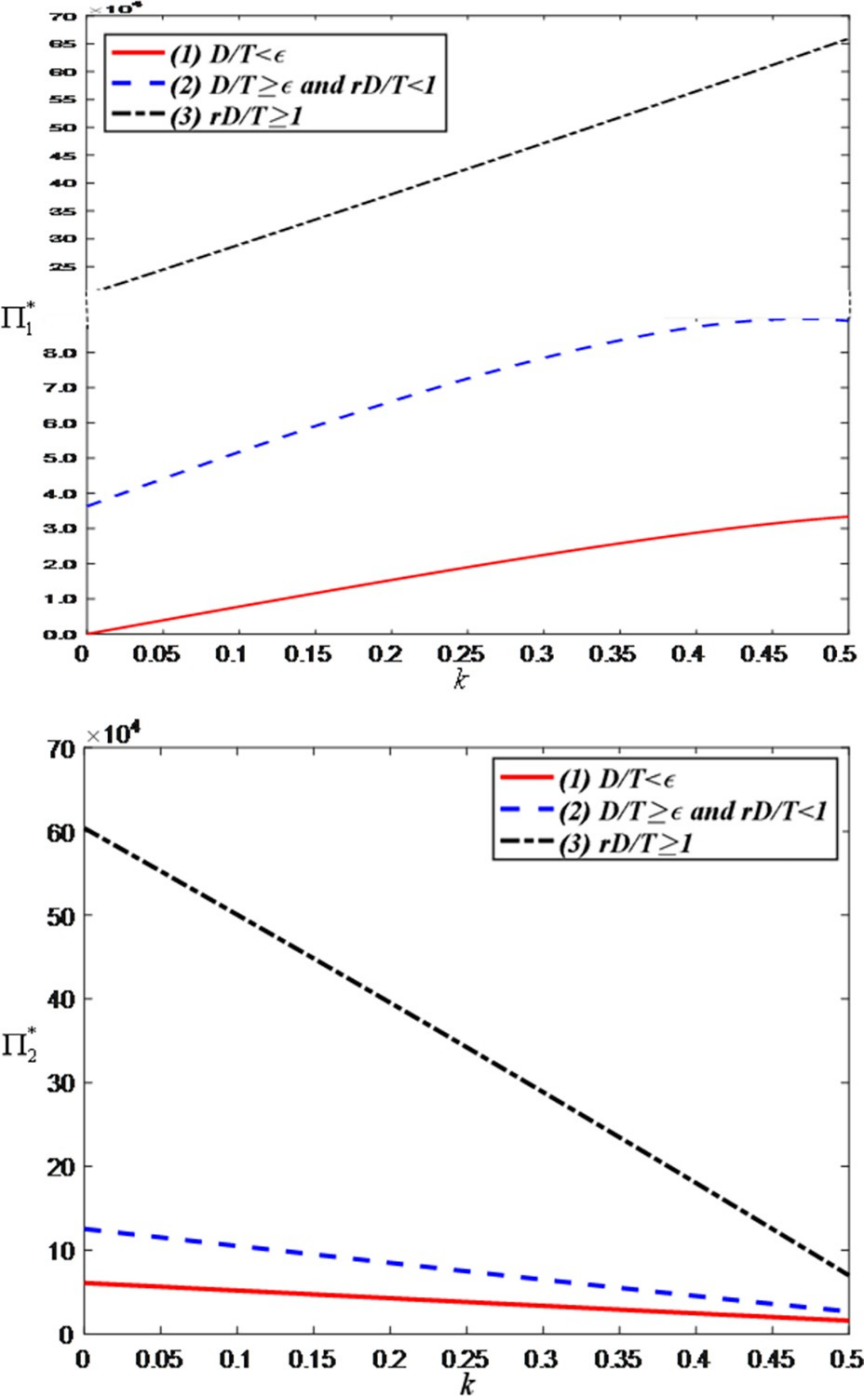
The impact of the cost coefficient of LS service effort on total profit. (a) Total profit of the anchor. (b) Total profit of the brand retailer.

Fig 8 shows that the optimal profits for both the anchor and the brand retailer decrease as the anchor’s LS service effort cost coefficient increases. This is evident as an increase in the effort cost coefficient leads the anchor to reduce their service effort, which in turn lowers demand and further decreases overall profits.

**Fig 9:**
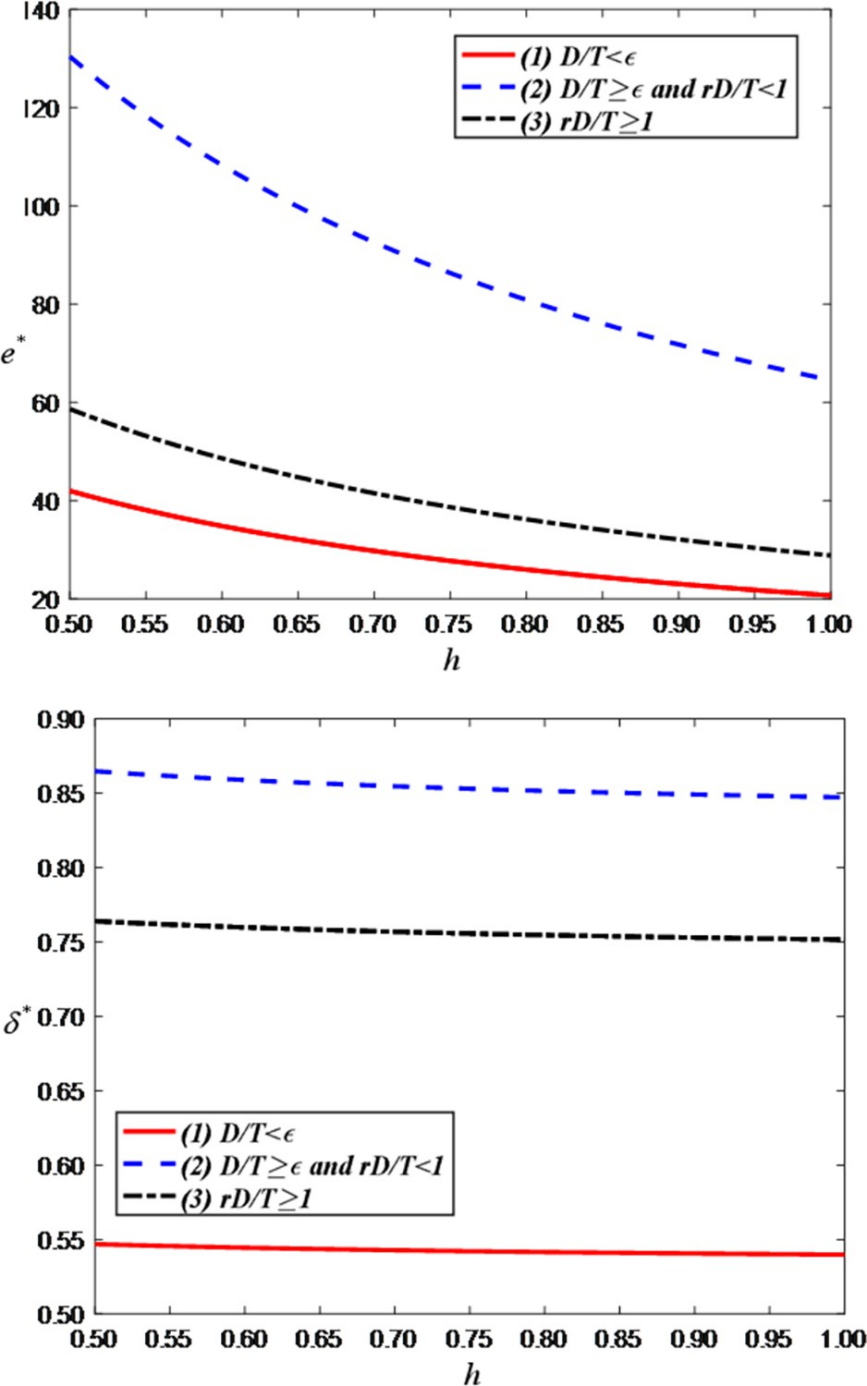
The impact of the ratio of confirmed-receipt sales volume to paid-successfully sales volume on price discount and LS service effort. (a) live streaming service effort. (b) price discount.

Thirdly, we examine the impact of the ratio of confirmed-receipt sales volume to paid-successfully sales volume on the optimal decisions and maximum profits and set the value range of *r* is [0.5, 1] to satisfy the condition 0<*ε*<*r*<1. Fig 9(A) shows that as the ratio of confirmed-receipt sales volume to paid-successfully sales volume increases, the LS service effort of the anchor increases. This is because a higher *r* indicates that consumers are more inclined to purchase goods rather than return them. To maintain or improve the receipt rate, anchors will increase their live streaming service effort to enhance the shopping experience and product quality. Similarly, as shown in Fig 9(B), as *r* increases, the price discount becomes larger. This is because brand retailers need to attract consumers to purchase by offering larger discounts. This strategy can help brand retailers gain more consumer recognition and sales in the fiercely competitive market.

**Fig 10:**
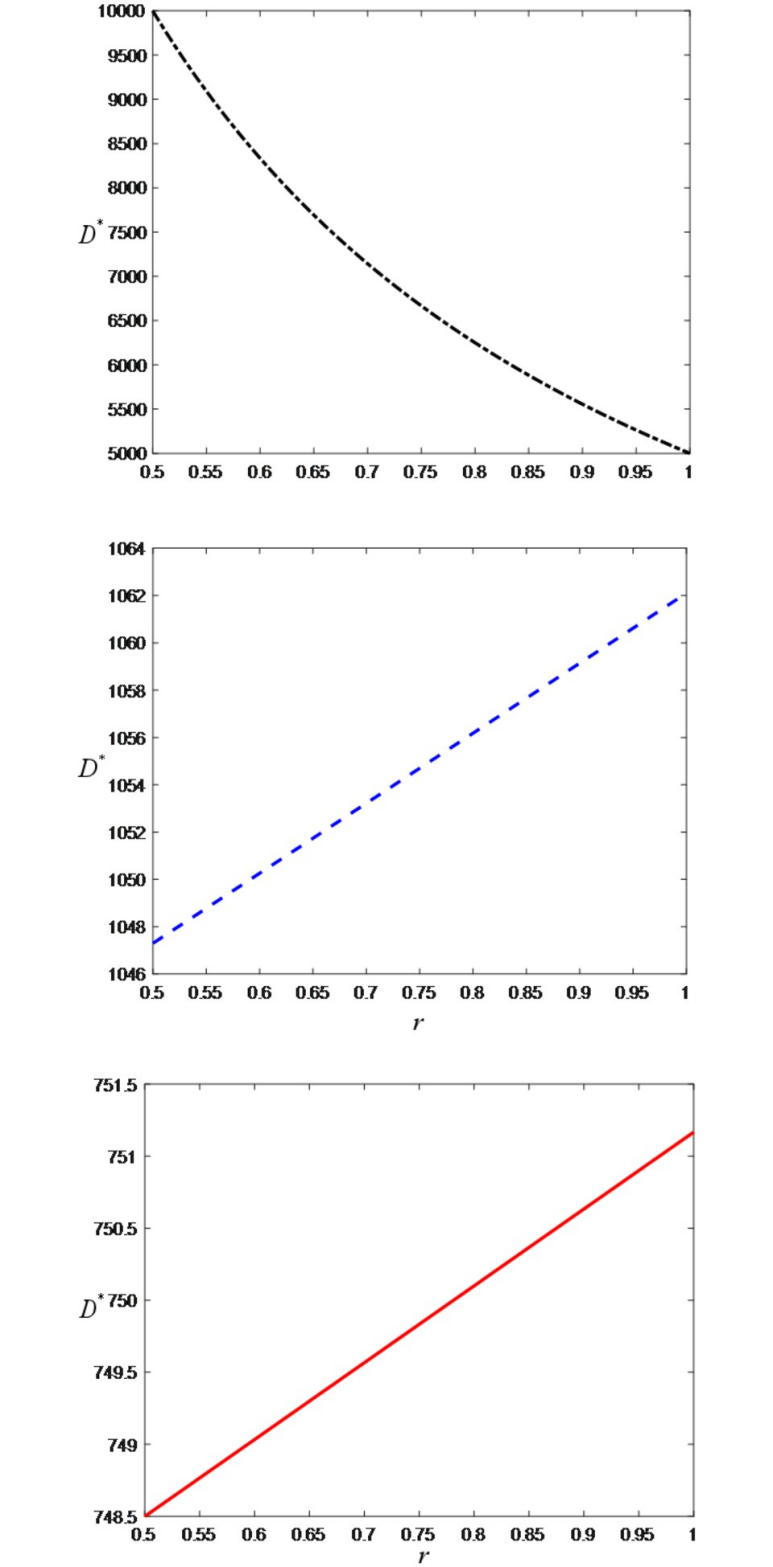
The impact of the ratio of confirmed-receipt sales volume to paid-successfully sales volume on optimal demand. (a) *D*/*T*<*ε*. (b) *D*/*T*≥*ε* and *rD*/*T*<1. (c) *rD*/*T*≥1.

Fig 10(A) and 10(B) show that the optimal demand increases with the increase in the ratio of confirmed-receipt sales volume to paid-successfully sales volume (i.e., retention rate) because a higher retention rate means that more purchasing actions are successfully converted into sales, reducing losses due to returns and increasing the overall demand. However, Fig 10(C) shows that when the demand reaches a certain threshold, the optimal demand increases with the increase of *r*. The reason is that as the retention rate increases, more potential demand can be converted into actual sales, thereby increasing the total demand.

**Fig 11:**
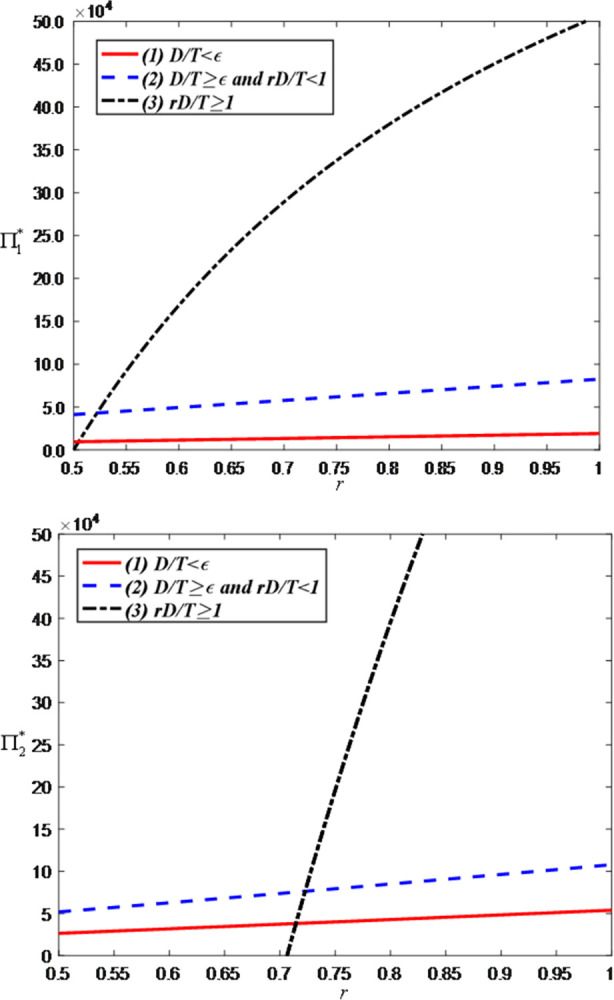
The impact of the ratio of confirmed-receipt sales volume to paid-successfully sales volume on total profits. (a) Total profits of the head anchor. (b) Total profits of the brand retailer.

Fig 11(A) and 11(B) demonstrates that as the ratio of confirmed-receipt sales volume to paid-successfully sales volume increases, the profits of both the anchor and brand retailers also increase. This is because a higher *r* indicates that consumers are more inclined to keep the purchased items rather than return them. A lower return rate helps reduce post-sale costs and losses since returns incur additional expenses such as shipping, handling, and restocking. Additionally, a higher receipt rate also reflects consumer satisfaction and trust in the product, which may encourage them to purchase more from the same brand or recommend the brand to others, thereby increasing brand loyalty and market share and consequently increasing the profits of both the anchor and brand retailers.

**Fig 12:**
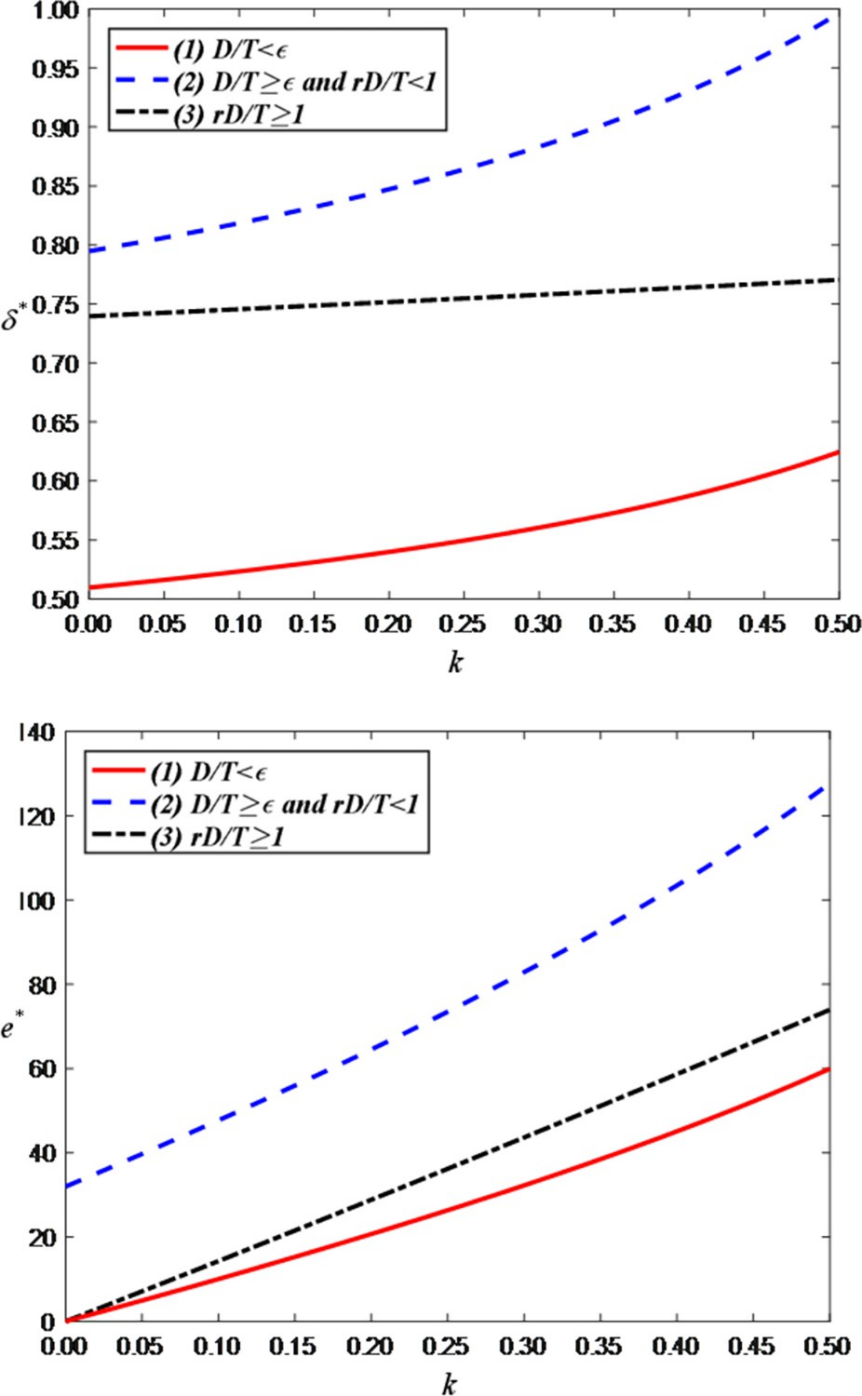
The impact of the sales commission rate on optimal decisions. (a) Price discount of the brand retailer. (b) LS service effort of the anchor.

Finally, we examine the impact of the sales commission rate on the optimal decisions and maximum profits and set the value range of *r* is [0, 0.5]. Fig 12(A) and 12(B) shows that as the sales commission rate increases, the brand’s price discount and the anchor’s efforts both increase. The reason behind this phenomenon is that higher commissions motivate anchors to put more effort into improving the quality of live streaming and interactions, thereby driving sales. In addition, since increasing commissions increases the brand’s costs, the brand can only retain higher product prices by increasing the discount rate.

**Fig 13:**
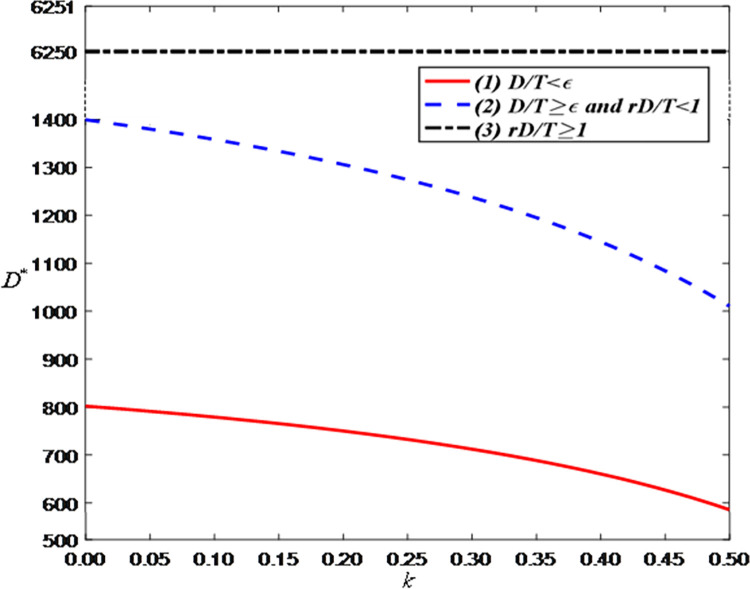
The impact of the sales commission rate on optimal demand.

Fig 13 shows that as the sales commission rate increases, consumer demand decreases. This is because the brand will improve the discount rate (i.e., raise product prices) to offset the increased costs, thereby reducing consumer purchase willingness. However, when the demand is greater than the threshold, the optimal demand has nothing to do with the sales commission rate.

**Fig 14:**
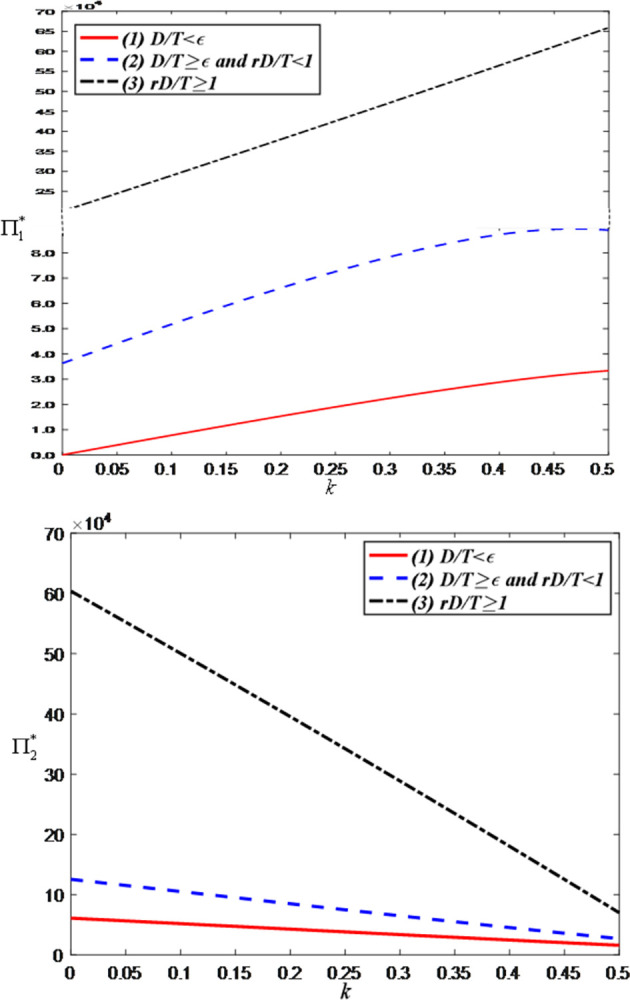
The impact of the sales commission rate on total profits. (a) Total profits of the anchor. (b) Total profits of the brand retailer.

Fig 14(A) and 14(B) shows that as the sales commission rate, the anchor’s profit increases while the brand’s profit decreases. This is because the increased commission directly enhances the anchor’s revenue but simultaneously raises the brand’s costs, resulting in a reduction in the brand’s profit.

## 6. Conclusions

As live streaming commerce gradually moves towards standardization, it becomes imperative to explore novel collaborative models to strengthen the partnership between brand retailers and anchors. This study investigates the live streaming commerce supply chain system composed of a brand retailer and an anchor, and establishes price discounts and live streaming service efforts decision models under the equal proportion settlement mode of pit fees for the brand retailer and anchor. Moreover, we assessed the profitability of both entities and conducted sensitivity analysis and numerical verification to validate the findings.

### 6.1. Discussion of findings

Our study reveals several significant insights under the equal proportion settlement mode.

#### (i) For the equal proportion settlement mode

Our study is based on the practice of live streaming commerce and is conducted within the framework of the equal proportion contract cooperation mode designed by Zhang and Xu [40]. While Zhang and Xu [40] focus on optimizing parameters such as the agreed sales volume, proportional threshold, and pit fees within the equal proportion settlement mode, our research treats these parameters as exogenous variables. Instead, we focus on the optimal decision-making between brand retailers and anchors under the equal proportion settlement mode. Therefore, our conclusions differ from those of Zhang and Xu [40]. For instance, we find that when the paid-successful sales volume is low, the optimal decisions of brand retailers and anchors exist, but they are independent of the ratio of the paid-successful sales volume to the agreed sales volume, as well as the agreed sales volume itself. When the paid sales volume is high, the pit fee positively influences optimal decisions. Moreover, when the ratio is greater than or equal to 1, the optimal demand is only affected by the ratio of the paid-successfully sales volume to agreed sales volume, as well as the agreed sales volume.

#### (ii) For brand retailers

Under the equal proportion settlement mode, when the paid-successfully sales volume is low, brand retailers are protected under the full refund pit fee settlement mode. When the paid-successful sales volume is high, the pit fee positively influences the optimal decisions of brand retailers. By offering larger price discounts to attract consumers, brand retailers can achieve higher profits. When the ratio of the paid-successfully sales volume to the agreed sales volume is greater than or equal to 1, the optimal demand is influenced only by this ratio, encouraging brand retailers to improve product quality to reduce return rates. Furthermore, the optimal price discount increases with the sensitivity of consumers to live streaming marketing, the anchor’s influence, and the consumers’ retention rate, and decreases with the cost-efficiency of LS service effort. Finally, the brand retailer’s optimal profits increase with the anchor’s influence and consumer retention rate and decrease with the increase in the cost efficiency of LS service effort and the sales commission rate.

#### (iii) For anchors

Under the equal proportion settlement mode, when the paid-successfully sales volume is low, anchors can only earn sales commissions. However, when the paid-successful sales volume is high, anchors should invest more in LS service efforts to gain a higher proportion of the pit fee, thereby boosting overall sales and profits. In addition, the optimal LS service effort increases with the consumer sensitivity to live streaming marketing, the anchor’s influence, and consumers retention rate, but decreases as the cost efficiency of LS service effort decreases. The anchor’s optimal profit increases with the anchor’s influence, consumer retention rate, and sales commission rate, while it decreases with the increase in the cost efficiency of LS service effort.

### 6.2. Management implications

Combining the equilibrium results and numerical analysis in this paper, we summarize the study’s management implications from the perspectives of brand retailers and anchors.

#### (i) Brand retailers

As the upper limit of pit fees constrains the incentive for anchors, brand retailers can consider adding agreement terms to provide extra rewards for anchors who exceed sales targets, further stimulating their motivation, driving sales growth, and promoting the stability and continuous development of cooperative relationships. In addition, in situations where the paid-successfully sales volume is high, brand retailers can attract consumers by offering greater price discounts, thereby enhancing the product’s value for money and increasing sales. Moreover, brand retailers can reduce return rates, lower after-sales costs, and enhance profits by increasing the ratio of confirmed-receipt sales volume to paid-successful sales volume. Therefore, brand retailers should actively implement measures to improve product consumer retention rate, thereby boosting customer satisfaction and trust. Finally, while appropriately increasing the sales commission rate can incentivize anchors to put in more effort and improve live streaming quality, brand retailers must balance costs and benefits and formulate reasonable commission strategies.

#### (ii) Anchors

The equal proportion settlement mode of pit fee provides protection for retailers, but it also exposes the relatively weaker live streaming commerce capabilities of anchors. Therefore, anchors should actively enhance their influence by creating innovative content and strengthening interactions to attract more fans and viewers, thereby increasing traffic and sales in the live streaming room. Additionally, when selecting products to promote, anchors should prioritize high-quality, high-value products to reduce return rates and increase the ratio of confirmed-receipt sales volume to paid-successful sales volume, thus boosting consumer satisfaction. Lastly, with an increase in sales commission rates, anchors should invest more effort into improving quality and interactivity. However, they must also pay attention to the brand retailer’s price discount strategies and coordinate their live streaming service efforts accordingly.

### 6.3. Limitations and future research

This study aims to provide the optimal price discount and live streaming service efforts for brand retailers and anchors under the new cooperative model of the equal proportion settlement of pit fees. Although our study has drawn some valuable conclusions, we acknowledge that our study has some limitations. Firstly, we only consider this particular cooperative model. Further research could compare existing cooperative models, such as pure commission settlement and commission plus fixed pit fee settlement.

In addition, we only considered the interaction and revenue distribution between brand retailers and anchors while ignoring the influence of intermediaries (such as MCN organization). Future research can be extended to multi-agent game models to explore the complex interactive relationship between brands, anchors, and MCNs so as to provide more comprehensive strategic recommendations.

Finally, our analysis is based on some specific parameter settings, which may vary in different market environments and product categories. For example, the price elasticity and consumer retention rate of different products may vary significantly. Therefore, future research can verify the universality of the model through case analysis and calibrate parameters in different market environments to provide a wider range of application references.

## Supporting information

S1 Appendix Appendix. (DOCX)

S1 Dataset Minimal data set. (DOCX)
