# Supplementary material for: Pricing and service effort strategy in live streaming commerce supply chain under the equal proportion settlement mode
Source: PLoS One. 2024 Aug 29;19(8):e0309371. doi: 10.1371/journal.pone.0309371 (PMC11361675; doi:10.1371/journal.pone.0309371)
Supplement: S1 Appendix — (DOCX) [file pone.0309371.s001.docx]

**Appendix**

**Proof of Proposition 1.** According to the inverse solution method, we first consider the decision-making stage of the head anchor team. We construct the Lagrange equation for the profit function of the anchor in problem (1) as follows:

$L_{1}\left(e,\mu_{1}\right)=\left[k\delta pr\left(1-\alpha\right)-\mu_{1}\right]\left[a-b\delta p+\lambda\left(i+\eta e\right)\right]-he^{2}/2+\mu_{1}\varepsilon T,\text{ }\mu_{1}\geq0$ (A-1)

Due to $\partial^{2}\Pi_{1}/\partial e^{2}=-h<0$, $\Pi_{1}$ is a strictly concave function with respect to $e$, and there is an optimal $e$ to maximize $\Pi_{1}$. According to the Kuhn-Tucker condition (A-2), we can obtain the optimal reaction function of $e$ with respect to $\delta$ is $e^{*}\left(\delta\right)=\left(1-\alpha\right)kr\delta p\eta\lambda/h$.

$\left\{\begin{matrix} e\cdot\frac{\partial L_{1}}{\partial e}=e\cdot\left[k\delta pr\left(1-\alpha\right)\eta\lambda-he-\mu_{1}\eta\lambda\right]=0 \\ \mu_{1}\cdot\frac{\partial L_{1}}{\partial\mu_{1}}=\mu_{1}\cdot\left[\varepsilon T-a+b\delta p-\lambda\left(i+\eta e\right)\right]=0 \\ \mu_{1}\geq0 \end{matrix}\right.$ (A-2)

Next, we consider the decision-making stage of the retailer. We construct the Lagrange equation for the profit function of the retailer in problem (1) as follows:

$L_{2}\left(\delta,\mu_{2}\right)=\left\{r\left[\left(1-k-\beta\right)\delta p-c\right]-\mu_{2}\right\}\left[a-b\delta p+\lambda\left(i+\eta e^{*}\left(\delta\right)\right)\right]+\mu_{2}\varepsilon T,\text{ }\mu_{2}\geq0$ (A-3)

Due to $\partial^{2}\Pi_{2}/\partial\delta^{2}=-2brp^{2}\left(1-k-\beta\right)<0$, $\Pi_{2}$ is a strictly concave function with respect to $\delta$, and there is an optimal $\delta$ to maximize $\Pi_{2}$. According to the Kuhn-Tucker condition (A-4), we can obtain the retailer’s optimal product price discount is $\delta^{*}=\frac{hA+cB}{2pB\left(1-k-\beta\right)}$, where $A=\left(a+i\lambda\right)\left(1-k-\beta\right)$, $B=hb-\left(1-\alpha\right)kr\eta^{2}\lambda^{2}$.

$\left\{\begin{matrix} \delta\cdot\frac{\partial L_{2}}{\partial\delta}=\delta\cdot\frac{\partial L_{2}}{\partial\delta}\left\{r\left[\left(1-k-\beta\right)\delta p-c\right]-\mu_{2}\right\}\left[a-b\delta p+\lambda\left(i+\eta e^{*}\left(\delta\right)\right)\right]=0 \\ \mu_{2}\cdot\frac{\partial L_{2}}{\partial\mu_{2}}=\mu_{2}\cdot\left\{r\left[\left(1-k-\beta\right)\delta p-c\right]-\mu_{2}\right\}\left[a-b\delta p+\lambda\left(i+\eta e^{*}\left(\delta\right)\right)\right]=0 \\ \mu_{2}\geq0 \end{matrix}\right.$ (A-4)

Substituting $\delta^{*}$ into $e^{*}\left(\delta\right)=\left(1-\alpha\right)kr\delta p\eta\lambda/h$, we can obtain the anchor’s optimal LS effort is $e^{*}=\frac{kr\lambda\eta\left(1-\alpha\right)\left(hA+cB\right)}{2hB\left(1-k-\beta\right)}$. Then, substituting $\delta^{*}$ and $e^{*}$ into $D$, $\Pi_{1}$, and $\Pi_{2}$, we can obtain $D^{*}=\frac{hA-cB}{2h\left(1-k-\beta\right)}$, $\Pi_{1}^{*}=\frac{kr\left(1-\alpha\right)\left(hA+cB\right)\left[hA\left(3B-bh\right)-cB(B+bh)\right]^{2}}{8h\left(1-k-\beta\right)^{2}B^{2}}$, and $\Pi_{2}^{*}=\frac{r\left(hA-cB\right)^{2}}{4hB\left(1-k-\beta\right)}$.

**Proof of Lemma 1.** According to the problem (1) and Proposition 1, we know  $\delta^{*}>0$, $e^{*}>0$, and $D^{*}<\varepsilon T$. Substituting $A=\left(a+i\lambda\right)\left(1-k-\beta\right)$ into $D^{*}=\frac{hA-cB}{2h\left(1-k-\beta\right)}$, we can obtain $D^{*}=\frac{h\left(a+i\lambda\right)\left(1-k-\beta\right)-cB}{2h\left(1-k-\beta\right)}<\varepsilon T$. Solving the inequality yields a range of parameters $i$ satisfying: $i<\frac{cB-\left(1-k-\beta\right)\left(a-2\varepsilon T\right)h}{\left(1-k-\beta\right)\lambda h}$.

**Proof of Proposition 2.** According to the inverse solution method, we first consider the decision-making stage of the head anchor team. We construct the Lagrange equation for the profit function of the anchor in problem (2) as follows:

$\left\{\begin{matrix} L_{1}\left(e,\mu_{1},\nu_{1}\right)=\left\{r\left[\left(1-\alpha\right)k\delta p+L/T\right]-\mu_{1}+\nu_{1}\right\}\left[a-b\delta p+\lambda\left(i+\eta e\right)\right]-he^{2}/2+\mu_{1}T/r-\nu_{1}\varepsilon T \\ \mu_{1}\geq0,\text{ }\nu_{1}\geq0 \end{matrix}\right.$ (A-5)

Due to $\partial^{2}\Pi_{1}/\partial e^{2}=-h<0$, $\Pi_{1}$ is a strictly concave function with respect to $e$, and there is an optimal $e$ to maximize $\Pi_{1}$. Using the Kuhn-Tucker condition, we can obtain $e^{*}\left(\delta\right)=\left[\left(1-\alpha\right)k\delta pT+L\right]r\eta\lambda/\left(hT\right)$ by solving the following equation set (A-6).

$\left\{\begin{matrix} \frac{\partial L_{1}}{\partial e}=0 \\ \mu_{1}\cdot\frac{\partial L_{1}}{\partial\mu_{1}}=0 \\ \nu_{1}\cdot\frac{\partial L_{1}}{\partial\nu_{1}}=0 \\ \mu_{1}\geq0,\text{ }\nu_{1}\geq0 \end{matrix}\right.$ (A-6)

Next, we consider the decision-making stage of the retailer. We construct the Lagrange equation for the profit function of the retailer in problem (2) as follows:

$\left\{\begin{matrix} L_{2}\left(\delta,\mu_{2},\nu_{2}\right)=\left\{r\left[\left(1-k-\beta\right)\delta p-c-L/T\right]-\mu_{2}+\nu_{2}\right\}\left[a-b\delta p+\lambda\left(i+\eta e^{*}\left(\delta\right)\right)\right]+\mu_{2}T/r-\nu_{2}\varepsilon T \\ \mu_{2}\geq0,\text{ }\nu_{2}\geq0 \end{matrix}\right.$ (A-7)

Due to $\partial^{2}\Pi_{2}/\partial\delta^{2}=-2brp^{2}\left(1-k-\beta\right)<0$, $\Pi_{2}$ is a strictly concave function with respect to $\delta$, and there is an optimal $\delta$ to maximize $\Pi_{2}$. Using the Kuhn-Tucker condition, the optimal product price discount $\delta^{*}=\frac{\left(hA+cB\right)T+\left(2B-bh\right)L+E}{2pBT\left(1-k-\beta\right)}$ can be obtained by solving the following equation set (A-8).

$\left\{\begin{matrix} \frac{\partial L_{2}}{\partial\delta}=0 \\ \mu_{2}\cdot\frac{\partial L_{2}}{\partial\mu_{2}}=0 \\ \nu_{2}\cdot\frac{\partial L_{2}}{\partial\nu_{2}}=0 \\ \mu_{2}\geq0,\text{ }\nu_{2}\geq0 \end{matrix}\right.$ (A-8)

Substituting $\delta^{*}$ into $e^{*}\left(\delta\right)=\left[\left(1-\alpha\right)k\delta pT+L\right]r\eta\lambda/\left(hT\right)$, we can obtain the anchor’s optimal LS effort is $e^{*}=\frac{r\eta\lambda\left(F+G-H\right)}{2hBT\left(1-k-\beta\right)}$. Similarly, by substituting $\delta^{*}$ and $e^{*}$ into $D$, $\Pi_{1}$, and $\Pi_{2}$, we can obtain the optimal demand or paid-successfully sales volume is $D^{*}=\frac{T\left(hA-cB\right)-bhL+E}{2hT\left(1-k-\beta\right)}$, the anchor’s maximum profit is $\Pi_{1}^{*}=\frac{r\left(H-F-G\right)\left[h\left(bL+bcT-TA\right)\left(3B-bh\right)+r\eta^{2}\lambda^{2}H+cT(bh-B)^{2}\right]}{8hT^{2}B^{2}\left(1-k-\beta\right)^{2}}$, and the retailer’s maximum profit is $\Pi_{2}^{*}=\frac{r\left[T\left(hA-cB\right)-bhL+E\right]^{2}}{4hBT^{2}\left(1-k-\beta\right)}$, where $E=rL\eta^{2}\lambda^{2}\left(1-\beta-\alpha k\right)$, $F=bhL\left(2-2\beta-k-\alpha k\right)$, $G=kT\left(1-\alpha\right)\left(hA+cB\right)$, $H=L\left(1-\beta-\alpha k\right)\left(bh-B\right)$.

**Proof of Lemma 2.** According to the problem (2) and Proposition 2, we know $\delta^{*}>0$, $e^{*}>0$, and $\varepsilon T\leq D^{*}<\frac{T}{r}$. Substituting $A=\left(a+i\lambda\right)\left(1-k-\beta\right)$ into $D^{*}=\frac{T\left(hA-cB\right)-bhL+E}{2hT\left(1-k-\beta\right)}$, and solving the inequality $\varepsilon T\leq D^{*}<\frac{T}{r}$ yields a range of parameters $i$ satisfying: $i<\frac{r\left(bhL+cBT-E\right)-\left(1-k-\beta\right)\left(ar-2T\right)hT}{\left(1-k-\beta\right)\lambda hrT}$ and $i\geq\frac{bhL+cBT-E-\left(1-k-\beta\right)\left(a-2\varepsilon T\right)hT}{\left(1-k-\beta\right)\lambda hT}$.

**Proof of Proposition 3.** According to the inverse solution method, we first consider the decision-making stage of the head anchor team. We construct the Lagrange equation for the profit function of the anchor in problem (3) as follows:

$L_{1}\left(e,\mu_{1}\right)=\left[rk\delta p\left(1-\alpha\right)+\mu_{1}\right]\left[a-b\delta p+\lambda\left(i+\eta e\right)\right]-he^{2}/2+L-\mu_{1}T/r,\text{ }\mu_{1}\geq0$ (A-9)

Due to $\partial^{2}\Pi_{1}/\partial e^{2}=-h<0$, $\Pi_{1}$ is a strictly concave function with respect to $e$, and there is an optimal $e$ to maximize $\Pi_{1}$. Using the Kuhn-Tucker condition, we can obtain $e^{*}\left(\delta\right)=\left(1-\alpha\right)kr\delta p\eta\lambda/h$ by solving the following equation set (A-10).

$\left\{\begin{matrix} \frac{\partial L_{1}}{\partial e}=0 \\ \mu_{1}\cdot\frac{\partial L_{1}}{\partial\mu_{1}}=0 \\ \mu_{1}\geq0 \end{matrix}\right.$ (A-10)

Next, we consider the decision-making stage of the retailer. We construct the Lagrange equation for the profit function of the retailer in problem (3) as follows:

(A-11)

Due to $\partial^{2}\Pi_{2}/\partial\delta^{2}=-2brp^{2}\left(1-k-\beta\right)<0$, $\Pi_{2}$ is a strictly concave function with respect to $\delta$, and there is an optimal $\delta$ to maximize $\Pi_{2}$. Using the Kuhn-Tucker condition, the optimal product price discount $\delta^{*}=\frac{hI}{prB}$ can be obtained by solving the following equation set (A-12), where $I=r\left(a+i\lambda\right)-T$.

$\left\{\begin{matrix} \frac{\partial L_{2}}{\partial\delta}=0 \\ \mu_{2}\cdot\frac{\partial L_{2}}{\partial\mu_{2}}=0 \\ \mu_{2}\geq0 \end{matrix}\right.$ (A-12)

Substituting $\delta^{*}$ into $e^{*}\left(\delta\right)=\left(1-\alpha\right)kr\delta p\eta\lambda/h$, we can obtain the anchor’s optimal LS effort is $e^{*}=\frac{\left(1-\alpha\right)k\eta\lambda I}{B}$. Similarly, by substituting $\delta^{*}$ and $e^{*}$ into $D$, $\Pi_{1}$, and $\Pi_{2}$, we can obtain the optimal demand or paid-successfully sales volume in LS room is $D^{*}=\frac{T}{r}$, the anchor’s maximum profit is $\Pi_{1}^{*}=\frac{2rLB^{2}+hkI\left(1-\alpha\right)\left(2BT-bhI+IB\right)}{2rB^{2}}$, and the retailer’s maximum profit is $\Pi_{2}^{*}=\frac{hT\left[T\left(1-\beta-k\right)-rA\right]-rB\left(L+cT\right)}{rB}$.

**Proof of Lemma 3.** According to the problem (3) and Proposition 3, we know $D^{*}\geq\frac{T}{r}$, and if $I=r\left(a+i\lambda\right)-T>0$, $\delta^{*}>0$, $e^{*}>0$,. Thus, we can obtain $i>\frac{T-ra}{r\lambda}$.

**Proof of Corollary 1.** (1)According to Proposition 1, when the ratio of paid-successfully sales volume to agreed sales volume is less than $\varepsilon$, $\delta^{*}=\frac{hA+cB}{2pB\left(1-k-\beta\right)}$ and $e^{*}=\frac{kr\lambda\eta\left(1-\alpha\right)\left(hA+cB\right)}{2hB\left(1-k-\beta\right)}$, the first order partial derivatives and their positive or negative directions of $\delta^{*}$ and $e^{*}$ with respect to $\lambda$, $i$, $\eta$, $r$, and $h$ are as follows:

$\frac{\partial\delta^{*}}{\partial\lambda}=\frac{b\left[bhi\lambda+\left(2a+i\lambda\right)\left(bh-B\right)\right]}{2p\lambda B^{2}}>0$， $\frac{\partial\delta^{*}}{\partial i}=\frac{h\lambda}{2pB}>0$，$\frac{\partial\delta^{*}}{\partial\eta}=\frac{h\left(a+i\lambda\right)\left(bh-B\right)}{p\eta B^{2}}>0$，$\frac{\partial\delta^{*}}{\partial r}=\frac{h\left(a+i\lambda\right)\left(bh-B\right)}{2prB^{2}}>0$， $\frac{\partial\delta^{*}}{\partial h}=\frac{-\left(a+i\lambda\right)\left(bh-B\right)}{2pB^{2}}<0$，$\frac{\partial e^{*}}{\partial\lambda}=\frac{kr\eta\left(1-\alpha\right)\left[bh^{2}\left(A+bc\right)+ah\left(1-k-\beta\right)\left(bh-B\right)-c\left(bh^{2}-B^{2}\right)\right]}{2hB^{2}\left(1-k-\beta\right)}>0$， $\frac{\partial e^{*}}{\partial i}=\frac{bh-B}{2\eta B}>0$，$\frac{\partial e^{*}}{\partial\eta}=\frac{kr\lambda\left(1-\alpha\right)\left[Ah\left(2bh-B\right)+cB^{2}\right]}{2h\left(1-k-\beta\right)B^{2}}>0$，$\frac{\partial e^{*}}{\partial r}=\frac{k\eta\lambda\left(1-\alpha\right)\left(bh^{2}A+cB^{2}\right)}{2h^{2}B^{2}\left(1-k-\beta\right)}>0$， $\frac{\partial\delta^{*}}{\partial h}=\frac{-kr\eta\lambda\left(1-\alpha\right)\left(bh^{2}A+cB^{2}\right)}{2h^{2}B^{2}\left(1-k-\beta\right)}<0$.

According to Proposition 2, when the ratio of paid-successfully sales volume to agreed sales volume is greater than $\varepsilon$, and the ratio of confirmed-receipt sales to agreed sales volume is less than 1, $\delta^{*}=\frac{\left(hA+cB\right)T+\left(2B-bh\right)L+E}{2pBT\left(1-k-\beta\right)}$ and $e^{*}=\frac{r\eta\lambda\left(F+G-H\right)}{2hBT\left(1-k-\beta\right)}$, the first order partial derivatives and their positive or negative directions of $\delta^{*}$ and $e^{*}$ with respect to $\lambda$, $i$, $\eta$, $r$, and $h$ are as follows:

$\frac{\partial\delta^{*}}{\partial\lambda}=\frac{hr\eta^{2}\lambda\left[\left(a+i\lambda\right)J+2bL\right]+bh^{2}iT}{2pTB^{2}}>0$，$\frac{\partial\delta^{*}}{\partial\eta}=\frac{hr\eta\lambda^{2}\left[bL+J\left(a+i\lambda\right)\right]}{pTB^{2}}>0$， $\frac{\partial\delta^{*}}{\partial i}=\frac{h\lambda}{2pB}>0$，$\frac{\partial\delta^{*}}{\partial r}=\frac{h\eta^{2}\lambda^{2}\left[bL+J\left(a+i\lambda\right)\right]}{2pTB^{2}}>0$， $\frac{\partial\delta^{*}}{\partial h}=\frac{-r\eta^{2}\lambda^{2}\left[bL+J\left(a+i\lambda\right)\right]}{2pTB^{2}}<0$，$\frac{\partial e^{*}}{\partial\lambda}=\frac{r\eta\left\{B^{2}\left[cJ+L\left(1-\alpha k-\beta\right)\right]+h\left(1-k-\beta\right)\left[\left(bL+aJ\right)\left(2bh-B\right)+2bhi\lambda J\right]\right\}}{2hTB^{2}\left(1-k-\beta\right)}>0$， $\frac{\partial e^{*}}{\partial\eta}=\frac{r\lambda\left\{bhk\left(1-\alpha\right)\left[Th\left(bc+2A\right)+L\left(bh-B\right)\right]+bhF-B\left(hkTA+H\right)-cJ\left(b^{2}h^{2}-B^{2}\right)\right\}}{2hTB^{2}\left(1-k-\beta\right)}>0$，$\frac{\partial e^{*}}{\partial i}=\frac{kr\eta\lambda^{2}\left(1-\alpha\right)}{2B}>0$，$\frac{\partial e^{*}}{\partial r}=\frac{\eta\lambda\left\{bhF+bh^{2}J\left(A+bc\right)-\left(3bh-B\right)\left[cJ\left(bh-B\right)+H\right]\right\}}{2hTB^{2}\left(1-k-\beta\right)B^{2}}>0$， $\frac{\partial\delta^{*}}{\partial h}=\frac{-r\eta\lambda\left\{bhF+bh^{2}J\left(A+bc\right)-\left(3bh-B\right)\left[cJ\left(bh-B\right)+H\right]\right\}}{2h^{2}TB^{2}\left(1-k-\beta\right)B^{2}}<0$, where $J=\left(1-\alpha\right)kT$.

According to Proposition 3, when the ratio of confirmed-receipt sales volume to agreed sales volume is greater than or equal to 1, $\delta^{*}=\frac{hI}{prB}$ and $e^{*}=\frac{\left(1-\alpha\right)k\eta\lambda I}{B}$, the first order partial derivatives and their positive or negative directions of $\delta^{*}$ and $e^{*}$ with respect to $\lambda$, $i$, $\eta$, $r$, and $h$ are as follows:

$\frac{\partial\delta^{*}}{\partial\lambda}=\frac{b\left[bhi+k\eta^{2}\lambda\left(1-\alpha\right)\left(2I-ir\lambda\right)\right]}{pB^{2}}>0$， $\frac{\partial\delta^{*}}{\partial i}=\frac{h\lambda}{pB}>0$，$\frac{\partial\delta^{*}}{\partial\eta}=\frac{2hk\eta\lambda^{2}I\left(1-\alpha\right)}{pB^{2}}>0$，$\frac{\partial\delta^{*}}{\partial r}=\frac{h\left(bhI-BI+BT\right)}{pr^{2}B^{2}}>0$， $\frac{\partial\delta^{*}}{\partial h}=\frac{-k\eta^{2}\lambda^{2}I\left(1-\alpha\right)}{pB^{2}}<0$，$\frac{\partial e^{*}}{\partial\lambda}=\frac{k\eta\left(1-\alpha\right)\left(2bhI-arB+BT\right)}{B^{2}}>0$， $\frac{\partial e^{*}}{\partial i}=\frac{kr\eta\lambda^{2}\left(1-\alpha\right)}{B}>0$，$\frac{\partial e^{*}}{\partial\eta}=\frac{k\lambda I\left(1-\alpha\right)\left(2bh-B\right)}{B^{2}}>0$，$\frac{\partial e^{*}}{\partial r}=\frac{k\eta\lambda\left(1-\alpha\right)\left[bh\left(a+i\lambda\right)-J\eta^{2}\lambda^{2}\right]}{B^{2}}>0$， $\frac{\partial\delta^{*}}{\partial h}=\frac{-bk\eta\lambda I\left(1-\alpha\right)}{B^{2}}<0$, where $I=r\left(a+i\lambda\right)-T$.

Therefore, in the LS e-commerce supply chain with equal proportion settlement mode of “pit fee”, both $\delta^{*}$ and $e^{*}$ increase in $\lambda$, $i$, $\eta$, and $r$, and decrease in $h$.
