# Supplementary material for: Pricing and service effort strategy in live streaming commerce supply chain under the equal proportion settlement mode
Source: PLoS One. 2024 Aug 29;19(8):e0309371. doi: 10.1371/journal.pone.0309371 (PMC11361675; doi:10.1371/journal.pone.0309371)
Supplement: S1 Dataset — (DOCX) [file pone.0309371.s002.docx]

**Minimal data set**

We first verify the impact of key parameters on the optimal LS service effort of the anchor, optimal price discount in the LS room, optimal demand as well as the optimal total profit of the anchor and the brand retailer. Readers can replicate the results of our study through the proofs in the Appendix.

Given the numerous parameters involved in this study, we have chosen to conduct sensitivity analysis on the key parameters that significantly impact the equal proportion settlement mode. In practice, since e-commerce platforms typically charge a fixed commission of 5%, we exclude the commission paid by the brand retailer to the e-commerce platform from our analysis and focus only on the interaction factors between the brand retailer and the anchor. Additionally, product price and cost are generally confirmed by the brand retailer before cooperation, so these factors are also excluded from the sensitivity analysis. Similarly, the commission parameters between the MCN organization and the anchor are not considered. Ultimately, we selected the key $h$, $r$, $i$and $k$to examine their effects on the optimal decisions, demand, and profits of both the brand retailer and the anchor.

In numerical analysis of this paper, the original data of parameters are: $p=300$; $c=50$; $k=0.2$; $a=2000,3000,10000$; $b=8$; $\lambda=1$; $\alpha=0.2$; $\beta=0.05$; $L=200000$; $T=5000$; $\varepsilon=0.2$; $r=0.8$; $i=25$; $h=1$. Substituting these data to equations we obtained in the paper, the readers can redraw the figures in this paper.
